# Elucidating the role of compositional and processing variables in tailoring the technological functionalities of plant protein ingredients

**DOI:** 10.1016/j.crfs.2025.100971

**Published:** 2025-01-09

**Authors:** Lorenzo Barozzi, Stella Plazzotta, Ada Nucci, Lara Manzocco

**Affiliations:** a Department of Agricultural, Food, Environmental and Animal Sciences, University of Udine, Via Sondrio 2/a, 33100, Udine, Italy; b Lavazza innovation Center, Luigi Lavazza s.p.a., Str. di Settimo, 10156, Famolenta, Italy

**Keywords:** Protein-rich plants, Functional properties, Protein extraction, Protein purification, Protein flour, Protein concentrate, Protein isolate

## Abstract

Although various plant protein (PP) ingredients are available on the market, their application in foods is not trivial, and food companies are struggling to identify PP ingredients fitting the intended use. To fill this gap, abundant literature has appeared but data are hardly comparable due to the absence of a recognized classification of PP ingredients accounting not only for protein purity but also for the process history, and of standardised protocols for technological functionality assessment. In this review, a comprehensive analysis of comparable literature data was thus carried out to elucidate the effect of composition and processing variables on PP technological functionalities. The review presents four sections describing: (i) the approach followed for the construction of a database of PP ingredient functionalities; (ii) the composition and processing factors relevant to PP ingredients; (iii) PP ingredient functional properties and methods used for their determination; (iv) the effect of composition and processing factors on PP ingredient functionalities. This analysis showed legume proteins to present the highest solubility and interfacial properties while pseudocereal ones the highest water-holding capacity. Although pure ingredients show higher functionalities, non-protein components could contribute to interfacial properties. Alkaline extraction, isoelectric precipitation and freeze-drying is the process mostly used in academic research to obtain PP ingredients. However, other extraction, purification, and drying methods can be properly combined, resulting in specific PP ingredient functionalities. Overall, this review highlights that, besides protein purity and source, knowledge of the processing history is required to select PP ingredients with desired functionalities.

## Highlights

• A wide variety of plant protein ingredients (PPI) are appearing on the market.
• PPI can be classified based on protein purity and technological history.
• PPI functionalities tend to increase with protein purity.
• Protein denaturation and non-protein compounds impair PPI functionalities.
• Standardized methods for the functionality assessment of PPI are needed.

## Graphical abstract

**Figure:**
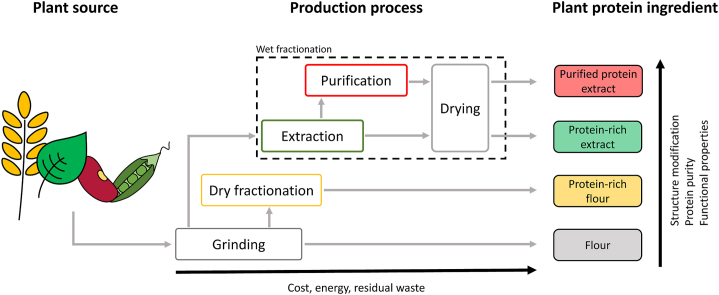

## 1 Introduction

The continuous growth of the population at global level is correlated to an increased demand for food, which, is estimated to rise by about 60% within 2050 (Wouters et al., 2016). In particular, the global demand for proteins cannot be satisfied by animal proteins alone (Foegeding, 2015; Pojić et al., 2018; Loveday, 2019). Animal food production has almost reached its maximum capacity besides being very impactful from the environmental point of view, due to the intensive consumption of soil, forage, and water, and high greenhouse gas emissions (Boland et al., 2013).

This situation has boosted the research for alternative protein sources such as insects, algae, fungi, and vegetable proteins (Ratnayake and Naguleswaran, 2022). Compared to animal proteins, plant proteins are more environmentally sustainable (Detzel et al., 2022). Replacing animal proteins with plant proteins would lead to 3–4 times lower land use and 30–40 times reduction of water use (Aiking, 2011). In addition, the consumption of plant proteins has been related to some nutritional advantages such as a low risk of cardiovascular diseases (Richter et al., 2015). The interest in this topic led to the birth of different multidisciplinary initiatives worldwide. Among the others, the “PROFETAS” program studied the environmental, technological, and societal feasibility of the transition from the currently predominant consumption of animal products to a society oriented towards plant foods (Profetas Program); Smart Protein, funded by Horizon 2020, aimed to develop alternative protein ingredients and foods with a positive impact on bio-economy, environment, nutrition, food security and consumer acceptance (Smart Protein); Plant Protein Enhancement Project, funded by FFAR, aims to understand the genetics and breeding of plant protein sources to uncover new plant protein crops and produce increases in yield, robustness and disease resistance (Plant Protein Enhancement Project); Sustainable Protein Production program, funded by 10.13039/501100000023Canadian Government, aims at developing tools and technology to improve the quality, safety and traceability of plan protein products (Sustainable Protein Production).

Evidence of environmental and health benefits of plant proteins consumption has stimulated a global discussion on the need for a progressive transition from animal to plant-based food, which is nowadays known as protein transition (Aiking and de Boer, 2020). The substitution of animal proteins with plant ones in food formulation is not trivial, since ingredient replacement requires the knowledge of multiple aspects related to safety (e.g., allergenicity), nutritional (e.g., digestibility, presence of antinutritional factors), and functional properties of the plant-based ingredient. The first author who defined protein functional properties was Kinsella (1976). According to this author, functional properties refer to “*any physicochemical property which affects the processing and behavior of protein in food systems, as judged by the quality attributes of the final product. These reflect complex interactions between the composition, structure, conformation, physicochemical properties of the proteins per se, other food components, and the nature of the environment in which these are associated or measured*”. Based on this definition, protein functional properties include a broad number of properties, from sensory to hydration, surface, and textural ones (Kinsella, 1976). Since then, the term functional properties has been used to include further protein properties such as nutritional and biological activities (Foegeding and Davis, 2011). Today, literature mainly uses the term functional properties to indicate protein technological behavior, while separately referring to sensory, nutritional and biological protein activities. Accordingly, in this review, the term “functional properties” will be used to indicate protein technological properties, with focus on solubility, liquid (water and oil) holding capacity, and interfacial properties (foam and emulsifying) (Wouters et al., 2016; Kumar et al., 2022).

As a result of the increasing market demand for plant proteins, plenty of research papers have addressed the study of protein functional properties. In the last 10 years, more than 5000 papers can be found on Scopus reporting in the title and/or as keywords the combination of the words “plant proteins” and “functional properties”. However, noncomparable data are often reported in these papers, due to the lack of globally recognized methodologies for the assessment of protein functionalities. Thus, despite this intense research production, the performances of plant protein ingredients are still hard to predict. Moreover, a wide variety of plant protein ingredients is nowadays available on the market, each of them with a specific nature and technological history. In this regard, a number of different variables might impact the functional properties of plant protein ingredients, ranging from intrinsic factors (*i.e.*, protein source and purity) to processing (*i.e.*, extraction, purification, and drying conditions) ones.

The present review aims to elucidate the role of: (i) main compositional variables of plant protein ingredients and (ii) principal processing variables applied during preparation in tailoring their technological functionalities. To this aim, the attention was focused on the rational classification and comparison of plant protein ingredients based on compositional factors and processing conditions suffered during their preparation.

## 2 Methodology

This systematic review was designed following the PRISMA guidelines (Page et al., 2021), as shown in Supplementary Fig. 1. The research database Scopus was searched in January 2024. The search identified works published from January 1, 2000 to December 31, 2023. Only papers published in English were included, and only results within the following subject areas were considered: “agricultural and biological science”, “chemistry”, “environmental science” and “material science”. The following search words were entered into the Scopus database, selecting the search option “title, abstract, and keywords”: “plant proteins”, “technological properties”, “functional properties”, “physicochemical”, “extraction”, “alkaline extraction”, “salt extraction”, “micellar precipitation”, “cereals”, “pseudocereals”, “legumes”, “oilseeds”, “leaves”, “protein isolate”, “protein concentrate”, “drying”, “solubility”, and “emulsifying properties” (singular and plural form written as reported). Detailed keyword combinations are reported in Supplementary Table 1. A number (n) of 43,614 papers were found and exported in an Excel database. Duplicates were searched and excluded (n = 12,953 excluded), as well as document types different from articles, such as books, book chapters, and conference contributions (Supplementary Fig. 1) (n = 3963 excluded). Results were further screened by only including articles published by Elsevier, Springer, Taylor and Francis, Cell, ACS publications, Academic Press, and MDPI (n = 7691 excluded). After this selection, a total number of 19,007 articles were further screened based on the presence among the authors' keywords or index keywords of the search words shown in Supplementary Table 2, referring to different plant matrices which can be used to produce plant protein ingredients (e.g., pea, lentil, wheat, oat, almond) (n = 9735 excluded). The plant matrices were chosen based on the ones most used in recent reviews on the topic (Contreras et al., 2019; Day, 2013; Day et al., 2022; Franca-Oliveira et al., 2021; Kumar et al., 2021; Loveday, 2019; Villacís-Chiriboga et al., 2020). Within the remaining articles (n = 9272) only those presenting among the authors’ keywords or index keywords search words relevant to functional properties, were selected. In this stage, abbreviations were used to include multiple search words: “solub” (used to include solubility, soluble, or similar words), “emuls” (used to include emulsion, emulsification, emulsifying, or similar words), “foam” (used to include foam, foaming, foamability, or similar words), “hold” (used to include hold, holding, or similar words), “funct” (used to include functional, functionality, or other similar words). On the selected articles (n = 4451), the same abbreviations relevant to functional properties were also used to screen the article abstract and title. The remaining articles (n = 2415) were further screened to exclude papers in which plant proteins were subjected to fermentation or used in the preparation of specific products such as bread or meat analogues, or in which the main topic was plant protein interaction with polysaccharides and animal proteins. To this aim, the following abbreviations were searched in the titles, author and index keywords: “fermen” (used to include fermented, fermentation, or other similar words), “microb” (used to include microbial, microbiological, or other similar words), “bacter” (used to include bacterial, or other similar words), “xylan” (used to include xylan, xylanose or other similar words), “pectin” (used to include pectin, pectinase, or other similar words), “agar” (used to include agar, agarose, or other similar words), “carrag” (used to include carragenin, carragenan, or other similar words), “dextr” (used to include dextrin, dextran, dextrose or other similar words), “conjug” (used to include conjugation, conjugate, or other similar words), “lacto” (used to include lactose, galactose, galactomannan or other similar words), “fung” (used to include fungi, fungal, or other similar words), “extru” (used to include extrusion, extruded or other similar words), “germin” (used to include germination, germinated or other similar words) and the words “meat”, “bread”, “interaction”, “starch”, “alginate”, “cellulose”, “polysaccharide”, “cow”, “sheep”, “goat”, “whey”, “milk”, “fish”, and “muscle”. The titles of the obtained articles (n = 1477) were screened one-by-one, followed by abstract screening to assess if they addressed the topic of the present review. Examples of excluded articles based on title were reported in Supplementary Table S3. As a result, 246 articles were finally selected to evaluate the effect of protein source and/or process on protein ingredient functional properties. These papers were selected and analyzed to identify the factors affecting the functional properties of plant protein ingredients. These factors were grouped into two main categories, *i.e.*, composition and processing factors (Table 1). The former refers to plant source and protein concentration in the ingredient (*i.e.*, protein purity), while processing variables included extraction, purification, and drying conditions applied for ingredient production (Joshi et al., 2015). These composition and processing factors were used as variables in the construction of a database on protein ingredient functionalities.

**Table 1:** Definition of variables related to composition and processing factors adopted for the development of the database on plant protein ingredient functionalities.

Factors		Definition
Composition	Plant source	Plant sources (e.g., legumes, oil seeds, cereals) used for ingredient production
	Protein purity	Protein weight concentration in the ingredient
Processing	Extraction	Technology used to extract the proteins from the plant source
	Purification	Technology used to isolate the proteins from co-extracted compounds
	Drying	Technology used to dry the protein ingredient

## 3 Composition and processing factors

### 3.1 Composition factors

#### 3.1.1 Plant source

For seed proteins, a widely accepted classification is the Osborne's one, which is based on protein extractability and solubility (Osborne, 1924). According to this classification, plant proteins can be divided into albumins, globulins, prolamins, and glutelins.

Along with seeds, also leaves are rich in proteins. Despite the extractability and solubility of leave proteins may lead to their classification as albumins and globulins (Rasheed et al., 2020), their common classification is not based on Osborne's one. Rather, leave proteins are commonly classified in white soluble, green unsoluble or cell membrane proteins (Tamayo Tenorio et al., 2016).

Based on these widely accepted plant protein classifications, in the present review, plant protein sources were classified as shown in Table 2. In particular, the classification distinguished between leaves and seeds and, among seeds, sub-categories were identified (i.e., cereals, pseudocereals, legumes, oilseeds, and shell fruit). Within each seed sub-category, sources share the botanical origin and/or a similar Osborne protein profile (Table 2). A similar categorization was already exploited in relevant literature (Loveday, 2019; Day et al., 2022; Zhang et al., 2024, Zhang et al., 2024).

**Table 2:** Average protein profile of different plant protein sources.

Source	Protein fraction	Content of specific protein fraction (g of specific protein fraction/100 g total protein)	Reference
Cereals	Glutelins	10–80	Ju et al. (2001); Hoogenkamp et al. (2017); Mäkinen et al. (2024); Taylor and Taylor (2024)
	Globulins	10–70	
	Prolamins	5–60	
	Albumins	5–20	
Pseudocereals	Albumins	20–50	Janssen et al. (2017)
	Globulins	10–50	
	Glutelins	10–40	
	Prolamins	0–10	
Legumes	Globulins	50–80	El Fiel et al. (2002); Nishinari et al. (2014); Rachwa-Rosiak et al. (2015); Hall et al. (2017); Lu et al. (2020); Amagliani et al. (2021)
	Glutelins	5–20	
	Albumins	0–20	
	Prolamins	0–5	
Oilseeds	Globulins	60–90	Aider and Barbana (2011); Amagliani et al. (2021)
	Albumins	10–20	
Shell fruit	Globulin	30–50	Li et al. (2018); Liu et al. (2018)
	Glutenin	30–40	
	Albumin	20–30	
Leaves	White soluble	30–40	Rasheed et al. (2020); Tamayo Tenorio et al. (2016)
	Green unsoluble	30–40	
	Cell membrane proteins	20–60	

Legumes, oilseed, and shell fruit protein profile is mainly represented by globulins (Hall et al., 2017; Li et al., 2018; Liu et al., 2018; Amagliani et al., 2021) while pseudocereals also contain a significant amount of albumins (Janssen et al., 2017). On the contrary, cereals mainly contain prolamins and glutelins (Janssen et al., 2017), except for oat, rich in globulins, while protein leaves can be divided into white soluble, green unsoluble, and cell membrane proteins, where the white soluble are mainly represented by enzymatic proteins, such as RuBisCo (Rasheed et al., 2020).

The different plant matrices within these sources are also characterized by different protein content, which range from 7 to 23 g/100 g dry matter in the case of cereals and pseudocereals, up to 40–50 g/100 g dry matter for legumes and oilseeds (Table 3).

**Table 3:** Average protein content of relevant plant matrices belonging to different sources.

Source	Plant matrix	Protein content (g protein/100 g dried plant matrix)	Reference
Cereals	Barley	10–20	Houde et al. (2018)
	Maize	9–12	Day (2013)
	Oat	17	Day et al. (2022)
	Rice	7–9	Day (2013)
	Wheat	8–15	Day (2013)
Pseudocereals	Amaranth	11–21	Shevkani et al. (2014); Day et al. (2022)
	Buckwheat	6–14	Day et al. (2022)
	Chia	19–23	López et al. (2018)
	Huauzontle	20	López-Monterrubio et al. (2020)
	Quinoa	12–23	Ruiz et al. (2016)
Legumes	Bean	16–26	Boye et al., 2010a, Boye et al., 2010b
	Chickpea	18–29	Boye et al., 2010a, Boye et al., 2010b
	Faba bean	23–26	Karaca et al. (2011b); Martínez-Velasco et al. (2018)
	Lentil	26–32	Boye et al., 2010a, Boye et al., 2010b
	Lupin	30–42	Lo et al. (2021)
	Pea	20–25	Lu et al. (2020)
	Soybean	35–40	Day (2013)
	Peanut	25	Joshi et al. (2015)
Oilseeds	Camelina	25–30	Boyle et al. (2018)
	Canola	21–32	Day et al. (2022); Karaca et al. (2011a)
	Cotton	23–25	Day et al. (2022)
	Flax	25	Karaca et al. (2011a)
	Hemp	20–30	Shen et al. (2021)
	Pepper	17–20	Wang et al. (2021)
	Sesame	14	Joshi et al. (2015)
	Sunflower	30–50	Dabbour et al. (2022)
Shell fruits	Almond	16–22	Sze-Tao and Sathe (2000)
	Brasilian nut	13–14	Joshi et al. (2015); Day et al. (2022)
	Cashew	18–19	Joshi et al. (2015); Day et al. (2022)
Leaves	Amaranth	23	Famuwagun et al. (2020)
	Eggplant	25	Famuwagun et al. (2020)
	Lablab	18–25	Zhao et al. (2021)
	Moringa	27	Cattan et al. (2022)
	Pumpkin	32	Famuwagun et al. (2020)

∗ calculated using total protein content as a reference.

∗∗g/100 g dried plant matrix.

#### 3.1.2 Protein purity

To date, there is no standardized categorization of protein ingredients based on protein purity. Nevertheless, the terms “concentrate” and “isolate” are of common use in the dairy sector to distinguish protein ingredients based on their purity, with “isolate” indicating purer derivatives than “concentrate” (Kelly, 2019). Similar to the dairy sector, from the same plant protein matrix, ingredients characterized by different protein purity can be obtained. In this regard, according to the protein content, relevant literature, reports the classification of plant protein ingredients in flours, concentrates, and isolates (Table 4).

**Table 4:** Protein content of protein flours, concentrates, and isolates according to different Authors.

Plant protein ingredient	Protein (%)	Reference
Protein flour	10–20	Akharume et al. (2021)
	10–30	Day (2013); Jiménez-Munoz et al. (2021)
	<65	Kumar et al. (2021)
	<50	Ma et al. (2022)
Protein concentrate	55–60	Akharume et al. (2021)
	>50	Day (2013); Jiménez-Munoz et al. (2021)
	65–90	Kumar et al. (2021)
	50–90	Ma et al. (2022)
Protein isolate	>80	Akharume et al. (2021)
	>80	Jiménez-Munoz et al. (2021)
	>90	Kumar et al. (2021)
	>90	Ma et al. (2022)

Even if it is commonly agreed that isolates are purer than concentrates and flours, the protein content discriminating against the different ingredients is ambiguous. Indeed, the values were arbitrarily defined by different Authors, resulting in overlapping values (Table 4). Furthermore, the term isolate is often used to describe ingredients obtained from intense purification processes aiming at reducing the concentration of non-protein compounds (Kumar et al., 2021). This process-driven definition further contributes to the ambiguity of the terms referring to protein ingredient purity.

Some Authors use the term “flours” to identify powder ingredients deriving from the simple drying and milling of the plant matrix, obtaining a protein content reflecting that of the plant matrix itself, ranging from 10 to 30 g/100 g dry matter (Table 3) (Day, 2013; Akharume et al., 2021; Jiménez-Munoz et al., 2021). By contrast, other Authors defined as flours all the ingredients with a protein content lower than the one typically associated with concentrates (i.e., <50%) (Kumar et al., 2021; Ma et al., 2022). According to this second definition, the term flour includes not only powders obtained by simple drying and milling, but also more concentrated powders obtained through dedicated process interventions such as dry fractionation (Allotey et al., 2022). The latter, however, may also lead to protein contents higher than 50–65%, in the typical range of concentrates (Allotey et al., 2022). This high discrepancy underlines that protein ingredients cannot be classified based on their protein content solely. Rather, the production process should be taken into consideration, shifting from a protein purity-based definition to a process-driven one.

### 3.2 Processing factors

**Fig. 1:**
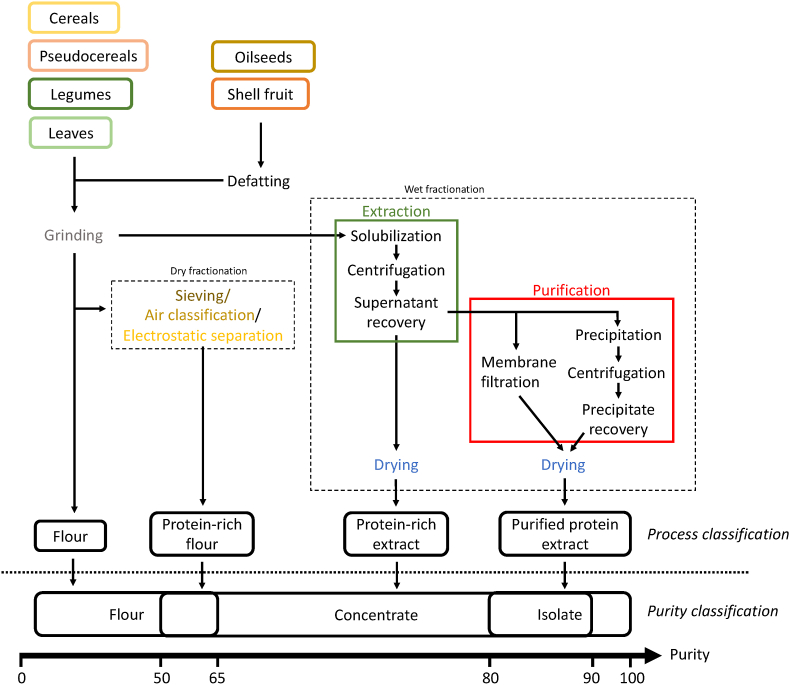
Key operations involved in the production process of protein ingredients characterized by increasing purity obtained from different plant sources.

Fig. 1 shows the key operations involved in the production of plant protein ingredients starting from the plant sources reported in Table 2. It can be observed that the application of deeply different processes can lead to ingredients with comparable protein purity (Fig. 1). Flours can be obtained by simple grinding or by dry fractionation approaches, while both dry and wet fractionation can be used to obtain concentrates; finally, purification techniques can be applied to produce ingredients with a purity within the range of both concentrates and isolates. Nevertheless, as discussed in the following, the production process highly impacts not only protein purity but also functionality, so that a classification based solely on protein content does not allow for a proper comparison among protein ingredients in terms of technological functionalities. For these reasons, in this review, a process-based classification of plant protein ingredients is proposed. The latter does not aim to replace the traditional purity-based one, but rather, to integrate it by taking into consideration the ingredient processing history.

#### 3.2.1 Flours

According to this classification, flours are obtained by grinding dried cereals, pseudocereals, legumes, oilseeds or shell fruits (Fig. 1). For each source, specific additional operation steps can be applied to remove undesired components. For example, in the case of cereals and legumes, the seeds can be dehusked or not to produce refined or whole flours, respectively (Papageorgiou and Skendi, 2018; Wood and Malcolmson, 2021). Oilseed flours are instead commonly produced from oil extraction cakes, which are preliminarily defatted to avoid defects deriving from lipid oxidation (Bárta et al., 2021). This process allows obtaining ingredients with concentrations up to 50% depending on the protein content of the plant matrix (Table 2). In other words, matrices particularly rich in proteins such as oilseed and legumes, can provide flours with higher values of protein, while, low-protein matrixes, such as cereals, will lead to low-protein flour.

#### 3.2.2 Protein-rich flours

To increase protein content, flours can be subjected to dry fractionation steps, which allows increasing protein purity up to 70% (Allotey et al., 2022), that is the protein content commonly associated with protein concentrates (Table 4). Dry fractionation includes air classification, sieving, and electrostatic separation, these technologies promote the separation of the protein-rich particles from the starch-rich ones exploiting their differences in density, dimension, or tribocharging behaviors (Assatory et al., 2019; Tabtabaei et al., 2023). These operations are classified as mild processes, since not implying the use of water or organic solvents, and lead to a minimum amount of residual material to be disposed of (van der Goot et al., 2016; Lie-Piang et al., 2023). Thanks to the mild operative conditions, dry fractionation operations highly preserve the native structure of proteins (Assatory et al., 2019).

#### 3.2.3 Protein-rich extract

After preliminary operations and grinding, the application of a processing step devoted to the extraction of proteins from the plant source can be applied (Fig. 1). The extraction is based on protein solubilization, which can be obtained by applying different technologies, followed by centrifugation and supernatant recovery to collect the protein-rich soluble fraction. Since process involves the use of extraction solvents, it is known as wet fractionation (Allotey et al., 2022). The latter favors not only the solubilization of protein but also that of hydrosoluble compounds including fibers, carbohydrates, sugars, and salts, resulting in ingredients with a protein content in the range 50–80%, which falls within the purity of protein concentrates (Table 4). However, wet fractionation may result in ingredients with protein content also in the typical range of flours, confirming that the classification of protein ingredients based on their purity solely is often misleading. For instance, after wet fractionation of amaranth and hempseed, dried ingredients with a purity of around 60% were obtained (Das et al., 2021; Potin et al., 2022). Such protein content can be classified as flours but also as concentrate (Table 4).

**Fig. 2:**
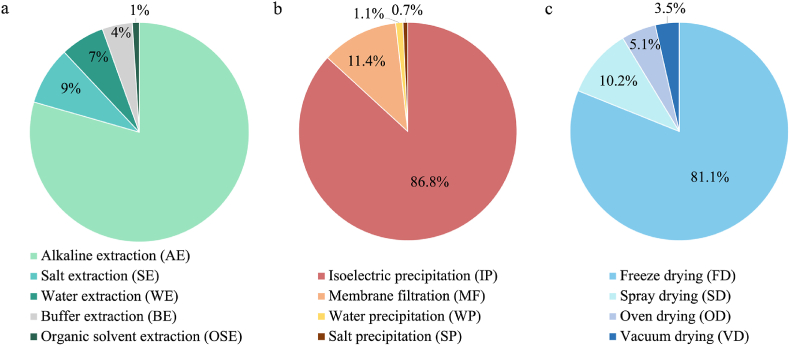
Cake charts relevant to the frequency of extraction (A), purification (B), and drying (C) technology used in the literature relevant to the production of plant protein-rich ingredients. Details regarding the data origin are reported in Supplementary Table S4.

Fig. 2A compares the different extraction technologies according to their frequency in the papers included in the database.

**Fig. 3:**
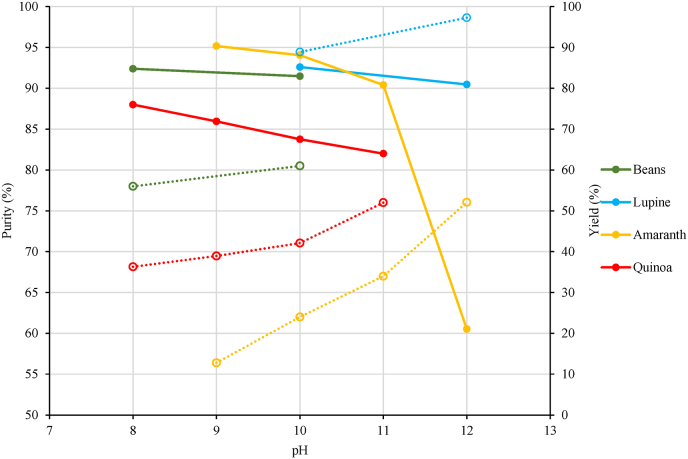
Purity (continuous lines) and yield (dotted lines) of purified protein extracts obtained from different plant sources through alkaline extraction at different pH values, followed by isoelectric precipitation. Details regarding the data origin are reported in Supplementary Table S4.

The technology most commonly applied in the literature to extract proteins from plant matrices is alkaline extraction (AE). This technology implies the contact of the ground plant matrix with alkaline aqueous solutions at mild temperatures (<70 °C). Under these conditions, the ionization of protein aminoacidic residues occurs, associated with the breakage of disulfide cross-linking and fiber solubilization. In this way, proteins are effectively released from the fibrous matrix (Hadidi et al., 2023). Since alkaline conditions allow for the solubilization of most plant proteins, AE represents a flexible technology, exploitable for the extraction of proteins from a wide variety of plant sources (Contreras et al., 2019). The application of alkaline conditions also leads to the solubilization of other components present in the plant matrix, mainly fibers. Nevertheless, by acting on the extraction pH, it is possible to modulate the presence of non-protein compounds in the final ingredient. Based on database analysis, pH 9.0 is the most diffused condition for performing AE (Shevkani et al., 2015; Yang et al., 2021). Some authors also used higher pH values, up to 12.0, to increase the extraction yield (Ngo and Shahidi, 2021). Fig. 3 shows the effect of the pH applied during AE on protein purity and yield of purified protein extracts obtained from different plant matrices.

The increase in pH results in a progressive increase of protein yield, since promoting protein solubilization. Nevertheless, these conditions concomitantly promote higher fiber extraction, thus decreasing protein purity in the final ingredient (Liu et al., 2023) (Fig. 3). In addition, extreme pH values also cause extensive changes in protein tertiary structure, favoring aggregation phenomena (Abugoch et al., 2008; Ruiz et al., 2016; Liu et al., 2023).

Water extraction (WE) at mild temperatures (<50 °C) allows the production of extracts particularly rich in albumins, the most hydrosoluble protein fraction. In this regard, Geerts et al. (2017) used this technology to produce albumin-rich extracts from peas. However, this technology is not widely applied in the literature relevant to plant protein ingredient production, accounting for about the 7% of the cases (Fig. 2A), most likely due to the low extraction yield as compared to less selective methodologies that concomitantly extract different protein fractions.

Extraction aided by salts (salt extraction, SE) and/or buffers (buffer extraction, BE) is generally less selective than AE and WE. In particular, SE allows obtaining ingredients containing not only water-soluble albumins but also globulins which, by definition, are salt-soluble. In this case, the plant matrices are dispersed in salt solutions containing sodium chloride, potassium chloride or other salt, even in combination, at concentrations in the range 0.5–0.8 M. These conditions were applied by Cordero-de-los-Santos et al. (2005) and Rodríguez-Ambriz et al. (2005) to produce protein ingredients from amaranth. The addition of a buffer (BE) may also increase protein extraction, thanks to the buffer cell-lysis action. For example, Tang (2007) used a Tris-HCl buffer to increase protein extraction from buckwheat.

The application of AE, WE, SE, and BE has been also proposed in combination. For example, Amirshaghaghi et al. (2017) combined AE with Tris-HCl and buffered saline borate extraction to extract proteins from almonds, while proteins were extracted from camelina by Boyle et al. (2018) exploiting a combination of BE and SE. The AE associated with Tris HCl leads to a lower protein yield (37%) compared to AE alone and AE in association with buffered saline borate (51 and 56% respectively) (Amirshaghaghi et al., 2017).

Organic solvent extraction based on ethanol, butanol, or acetone may be used to obtain ingredients rich in proteins such as prolamins (Contreras et al., 2019; Kumar et al., 2021). In this regard, ethanol extraction is typically applied for the production of zein extracts from maize (Tan et al., 2022).

#### 3.2.4 Purified protein extracts

When higher protein content is required, additional purification steps are applied after extraction, leading to purified protein extracts (Fig. 1), with concentrations in the range of 60–95% (Ge et al., 2021; Peyrano et al., 2016; Salcedo-Chávez et al., 2002; Shevkani et al., 2014), associated with the purity-based definition of both concentrates and isolates (Table 4).

Purification aims at increasing the protein content by acting on the supernatant obtained during extraction (Fig. 1). However, the remotion of soluble-co extracted compounds is particularly challenging and the operations applied to obtain highly pure ingredients mostly lead to low protein yields (Kumar et al., 2021). Fig. 2B classifies the purification technologies according to their frequency in the literature. The technologies applied for the purification can exploit the use of a membrane to separate the proteins from the other co-extracted compounds or induce the precipitation of the proteins and their recovery through centrifugation (Fig. 1). Isoelectric precipitation (IP) is the most commonly applied technology and allows for precipitating the proteins by adjusting the pH of the supernatant at the isoelectric point (pI) of the target protein. At this pH value, the surface charge of the protein is null, leading to the formation of insoluble protein aggregates, which are commonly collected by centrifugation. Alternatively, the supernatant can be filtered by using membranes with a tailored cut-off, usually in the range 0.5–14 kDa, according to the dimension of the target proteins. Among membrane filtration (MF) technologies (Fig. 2B), dialysis (D), and ultrafiltration (UF) (or reverse osmosis) are the most applied.

Even if the extraction technologies presented in Fig. 2A can be theoretically combined with any purification technology (Fig. 2B), only few combinations are practically exploited. Table 5 reports the most common procedures applied to obtain purified protein extracts.

**Table 5:** Combination of extraction and purification technologies and name used in literature to identify the process.

Extraction	Purification	Common name of the overall process
Alkaline extraction	Isoelectric precipitation	Alkaline extraction/Isoelectric precipitation
Alkaline extraction	Membrane filtration	Alkaline extraction
Salt extraction	Isoelectric precipitation	Salt extraction
Salt extraction	Membrane filtration	Salt extraction
Salt extraction	Water precipitation	Micellar precipitation
Salt extraction	Salt precipitation	Salt in/Salt out
Water extraction	Isoelectric precipitation	Water extraction
Water extraction	Membrane filtration	Water extraction
Organic solvent extraction	Isoelectric precipitation	Solvent extraction
Buffer extraction	Isoelectric precipitation	Buffer extraction
Buffer extraction	Membrane filtration	Buffer extraction

It is evident from Table 5 that there is no standardized nomenclature for the processes applied for protein extraction and purification. For instance, when alkaline extraction and isoelectric precipitation are applied, both the procedures are given in the process definition. By contrast, “salt extraction” is a term commonly used to describe salt extraction followed by isoelectric precipitation or membrane filtration. For this reason, the correct identification of the production process is often particularly challenging. A possibility to overcome these issues could be to identify the production process with a complex self-explaining nomenclature in which both the extraction and purification phases are made explicit, as already applied in the case of alkaline extraction followed by isoelectric precipitation.

Among the different technologies, the combination of AE and IP (AE + IP), or SE followed by MF (commonly reported as “salt extraction”) are the main ones exploited in the literature for the production of purified protein extracts. AE can also be applied in combination with MF, more specifically with ultrafiltration, allowing the physical separation of low-dimension compounds from high-dimension proteins. IP is the most flexible purification technology as it is applicable after any extraction method due to the precipitation of proteins induced by pH. On the opposite, water and salt precipitation are purification technologies that are mostly used after SE. The former exploits the addition of water to the protein-rich salt solution, inducing the precipitation of the proteins due to salt dilution. The combination of SE and water precipitation is commonly referred to as micellar precipitation (MP). In the case of salt precipitation (SP), the salt concentration of protein-rich salt solution is further increased inducing protein precipitation, due to the water-binding activity of the salt ions (Ma et al., 2022). The combination of SE and salt precipitation is generally referred to as “salting-in-salting-out” and precipitate proteins are commonly collected by centrifugation. Water and buffer extraction can be either followed by IP or membrane filtration. Despite the difference in the purification phase, in literature the processes are ambiguously named “water extraction” and “buffer extraction”, similarly SE + IP and SE + MF are known as “salt extraction”.

#### 3.2.5 Drying technology

Independently on the protein purity in the final ingredient, protein-rich ingredients are most commonly commercialized as powders. For this reason, drying is a key step in their production. In the case of protein-rich flours, the drying step can precede or follow the grinding one, depending on the source. For example, legumes and cereals are mostly commercialized in the form of dried grains, which are ground to obtain flour and can be further turned into protein-rich flour by dry fractionation (Santos et al., 2022). By contrast, in the case of protein-rich extracts and purified protein extracts drying is applied to the protein-rich aqueous dispersion obtained by wet fractionation (Fig. 1).

Oven-drying (OD) at different pressure conditions requires low-cost equipment but high energy consumption during the drying process; at the same time, this technology generally leads to intense protein denaturation, due to the applied temperature conditions (>50 °C), even under vacuum (vacuum drying, VD) (Shen et al., 2021), promoting protein mobility and structural rearrangements. Moreover, the presence of liquid-vapor interfaces induces intense capillary tensions in the ingredient particles, leading to a collapsed powder (Manzocco et al., 2024; Timilsena et al., 2016). To avoid collapse and obtain highly porous powders freeze drying (FD) and spray drying (SD) can be applied (Özdemir et al., 2022). FD is the drying technology mostly used at lab-scale to produce protein ingredients and thus the one most frequently found in the literature (Fig. 2C). FD operates at low-temperature, which guarantees minimal changes in protein structure, when the process is carefully optimized with particular reference to the freezing rate. In fact, the formation of big and inhomogeneously distributed ice crystals upon low-rate freezing has reported to cause protein interaction in the formation of aggregates (Chang et al., 2005). However, at industrial scale, freeze-drying is very limitedly used for the production of plant protein ingredients, being SD the most diffused technique, thanks to the lower equipment and operational cost, and the lower production time (Burger et al., 2022). Despite the high temperature applied in the SD chamber, this technique results in reduced protein denaturation thanks to the short drying time and the sudden cooling effect of water evaporation (Shen et al., 2021; Timilsena et al., 2016). It is thus important to underline the mismatch between literature findings, mostly relevant to protein samples obtained by freeze-dried and market ingredients which are industrially produced by spray-drying.

#### 3.2.6 Effect of process on protein purity, protein structure, and use of natural resources

**Fig. 4:**
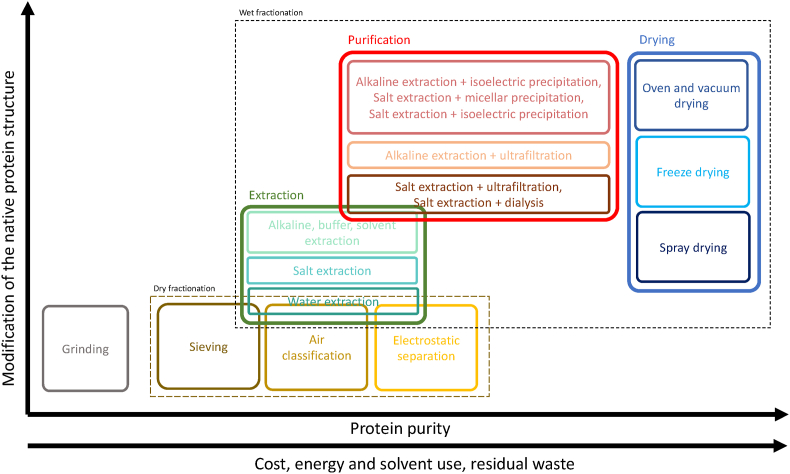
Comparison of production processes of protein ingredients in terms of protein purity, use of resources (cost, energy, solvent use), residual waste generation, and changes induced in protein native structure.

Fig. 4 reports the comparison of processes involved in the production of protein ingredients with different purity in terms of resource use (cost, energy, time), generation of residues, and change induced in protein native structure.

In the production of flour, grinding represents the main phase. Despite being characterized by minimal cost and energy consumption, this process leads to the production of different waste and by-products that only partially can be upcycled. After being produced, flour can be subjected to dry fractionation, in this case, the production of waste or by-products is extremely reduced since the flour is fractionated into protein-rich and starch-rich fractions which are both sold as ingredients. However, additional energy and costs are required for this process. Both flour and protein-rich flour production do not require solvent use and preserve the native protein structure.

Compared to dry fractionation, wet one is particularly energy-intensive and solvent-consuming (Fig. 4). Moreover, it is associated with the generation of a significant amount of polysaccharide-rich residues (van der Goot et al., 2016; Lie-Piang et al., 2023). Even if the latter can be properly treated to obtain value-added food ingredients, additional energy, time, costs, and solvents would required to pursue this zero-waste approach (Mondor and Hernández-Álvarez, 2022).

As shown in Fig. 4, AE causes a significant change in protein native structure. In particular, AE has been shown to promote protein unfolding with the exposure of hydrophobic and free sulfhydryl groups, leading to protein aggregation (Momen et al., 2021). SE and WE better preserve the original structure of the proteins (Fig. 4), due to the minimal structural changes induced by the ionic concentrations commonly used, and the absence of denaturing agents, respectively (Boyle et al., 2018). The use of both solvents and buffers leads to protein denaturation (Fig. 4), due to the rearrangement of protein structure as a response to modified environment polarity (Sze-Tao and Sathe, 2000; Amirshaghaghi et al., 2017).

The combination of the different extraction and purification technologies has an impact on protein structure (Fig. 4). In particular, AE + IP commonly causes significant protein denaturation and the formation of aggregates, due to the null net charge of the proteins at the IP, favoring protein-protein interactions (Liu et al., 2023). However, these denaturation effects can be reduced by applying mild extraction pH values (8–9) (Yang et al., 2021). By contrast, SE associated with ultrafiltration or dialysis (SE + UF, SE + D) has a lower impact on protein structure, since relying on protein separation aided by physical methods (Stone et al., 2015; Yang et al., 2021) (Fig. 4). Membrane technologies can also be combined with AE, as reported by Yang et al. (2021) in the production of purified protein extract from pea, resulting in intermediate structure alteration (Fig. 4).

## 4 Technological functionalities of plant proteins

### 4.1 Methodologies for the assessment of technological functionalities

Recent literature widely addressed the definition of plant protein functional properties as well as the analytical methods used for their determination. Different works deeply discuss the solubility of proteins (Gao et al., 2024; Grossmann and McClements, 2023), their role in promoting the formation of foams and emulsions (Foegeding and Davis, 2011; Amagliani et al., 2021; Schmitt et al., 2021), and the techniques involved in the study of functional properties (Wouters et al., 2016; Zhang et al., 2021; Kumar et al., 2022; Ma et al., 2022). In the following paragraphs, a brief definition of each functional property is provided, along with a description of the assays most commonly used for their assessment and the indexes used for their quantification.

#### 4.1.1 Solubility

Plant proteins are traditionally grouped according to their solubility in different media, which is the basis of Osborne classification (Osborne, 1924). However, from a technological point of view, water solubility is commonly assessed, due to the practical relevance of water as a food ingredient.

Water solubility of plant proteins can be defined as the ability of proteins to dissolve in an aqueous medium forming a homogeneous solution. The fundamental behind the solubility of proteins is their thermodynamic equilibrium in an aqueous solution, which mainly relies on the equilibrium between protein–protein and protein–solvent interactions at a given pH, ionic strength, and temperature (Gao et al., 2024).

According to (Gao et al., 2024), two main types of protein solubility methods are used in the literature. The thermodynamic ones are based on the determination at the equilibrium of the concentration of solubilized proteins and their insoluble crystals. However, these methods require sophisticated instruments, extreme protein purity, and long times (Gao et al., 2024). For this reason, kinetic methods are the most diffused, which analyze protein solubility, independently of protein purity, over a short time. The most commonly applied method is based on the dispersion overnight of the protein ingredient in an aqueous medium, followed by centrifugation and evaluation of the protein content either in the supernatant or in the pellet, using chemical (e.g., Kjeldhal method) or spectrophotometric assays (e.g., bicinchoninic acid assay - BCA). Protein solubility is then usually calculated according to equation (1):

(eq. 1) $$Solubilty\;\left(\%\right)=\frac{protein\;content\;in\;the\;supernatant}{total\;protein\;content\;of\;the\;dispersion}\;\cdot 100$$

Despite the operational simplicity, it must be underlined that protocols reported in the literature for solubility assessment are often noncomparable due to differences in terms of solubilization time and temperature, ionic strength, and pH of the aqueous medium, as well as centrifugation rate (Gao et al., 2024). Furthermore, variations in the expression of numerator and denominator of Equation (1) make the comparison of the results even more difficult.

#### 4.1.2 Interfacial properties

Interfacial properties identify the ability of protein ingredients to form and stabilize foams and emulsions. Plant protein are amphiphilic in nature, being able to act as surfactants, by decreasing surface tension, and adsorbing at the interface between water and air or water and oil, and stabilizing it (Schmitt et al., 2021; Zhang et al., 2021; Kumar et al., 2022).

Foaming properties are usually determined by whipping an aqueous suspension of the selected protein ingredient, followed by the evaluation of the formed foam volume and its stability over time. From this simple empiric test, foam capacity (FC) and foam stability (FS) are estimated (eqs. (eq. 2), (eq. 3))).

(eq. 2) $$FC\;\left(\%\right)=\frac{V_{F}}{V_{S}}\;\cdot 100$$

(eq. 3) $$FS\;\left(\%\right)=\frac{V_{t}}{V_{F}}\;\cdot 100$$

where *V*_*S*_*, V*_*F*_*,* and *V*_*t*_ (mL) are the volumes respectively of the initial suspension, of the formed foam immediately after whipping, or after a defined time.

Similar to foaming properties, emulsifying ones are usually determined by preparing an O/W emulsion using the considered protein ingredient as the emulsifier. Results are commonly reported as emulsifying activity index (EAI, eq (4)) and emulsifying stability index (ESI, eq. (5)) (Zhang et al., 2021).

(eq. 4) $$EAI\;\left(m^{2}/g\right)=\frac{2\cdot 2.303\cdot A_{0}\cdot DF}{C\cdot \phi \;\cdot \left(1-\theta \right)\cdot 10^{4}}$$

(eq. 5) $$ESI\;\left(\min\right)=\frac{A_{0}}{A_{0}-A_{t}}\;\cdot \Delta t$$

Where $A_{0}$ is the absorbance at 500 nm immediately after the emulsification, DF the dilution factor, C the concentration of the protein solution (g/mL), ϕ the optical path (cm), θ the oil volume fraction, $A_{t}$ the absorbance after a certain time and Δt the time.

Alternatively, emulsifying capacity (EC, eq (6)) and stability (ES, eq. (7)), are calculated, after inducing emulsion separation through centrifugation and heating.

(eq. 6) $$EC\;\left(\%\right)=\frac{V_{1}}{V_{0}}\;\cdot 100$$

(eq. 7) $$ES\;\left(\%\right)=\frac{V_{2}\;}{V_{0}}\;\cdot 100$$

Where $V_{0}$ is the total volume, $V_{1}$ the volume of emulsified layer after the centrifugation, and $V_{2}$ the volume after heating at 80 °C and centrifugation.

Emulsification ability and emulsion stability can also be assessed by determining the particle diameter distribution of the prepared emulsion immediately after preparation and over time. Although not relying on indirect spectrophotometrical determinations, only a minor number of papers uses this method (Plazzotta et al., 2021; Ma et al., 2022).

Despite these common procedures and data elaboration, comparing the foaming and emulsifying properties of plant protein ingredients is quite challenging. Indeed, the papers included in the database report a wide variety of operational conditions for the determination of both foaming and emulsifying properties. For example, protein concentration in water varies from 0.1 to 3.0% (w/w or w/v); whipping or emulsification can be performed by using home mixers, lab high-speed or high pressure homogenizers. Moreover, the whipping or emulsification time is often not reported. Furthermore, the evaluation time of both foam and emulsion stability ranges between 10 and 120 min. Finally, differences in equations (eq. 2), (eq. 3), (eq. 4), (eq. 5), (eq. 6), (eq. 7)) are often reported.

#### 4.1.3 Water and oil holding capacity

Water (WHC) and oil (OHC) holding capacity are defined as the amount of water or oil that can be held by 1 g of the plant protein ingredient (Zayas, 2012; Wouters et al., 2016). WHC and OHC are the resultant of physical and chemical interactions between the fluid and the protein ingredient. Indeed, these properties quantify both the fluid that is physically trapped in the pores of the protein ingredient, driven by capillary forces (Kumar et al., 2022), and the type and charge of amino acid residues exposed to the environment (Wouters et al., 2016). Hydrophilic residues increase WHC, while nonpolar side chains increase the OHC (Kaur and Singh, 2005; Kumar et al., 2022).

To determine WHC and OHC, a known amount of protein ingredient (*W*_1_) is added with excess water or oil and the mixture is centrifuged at 3000–15000 g, allowing for the removal of non-held fluid. The weight of the sediment (*W*2) is then used to calculate the amount of held water or oil (eq. (8)).

(eq. 8) $$WHC,OHC\;\left(g/g\right)=\frac{{W2-W}_{1}}{W_{1}}\;\cdot 100$$

Different from methods used for solubility and interfacial property assessment, this procedure, as well as the equation used to calculate WHC and OHC, is highly standardized in the literature relevant to the assessment of these functionalities of plant protein ingredients.

## 5 Effect of composition and processing variables on technological functionalities of plant protein ingredients

### 5.1 Effect of composition on protein technological functionalities

**Fig. 5:**
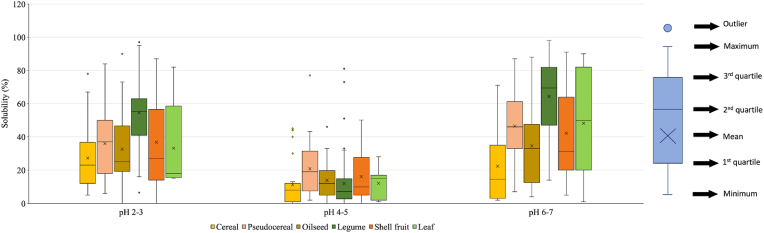
Protein solubility at different pH of plant protein ingredients obtained by alkaline extraction followed by isoelectric precipitation. At least 5 solubility values were considered for each plant source. Solubility is expressed according to equation (1), as the percentage of proteins solubilized upon kinetic determination, as compared to total proteins in the ingredient. Details regarding the data origin are reported in Supplementary Table S4.

Since extraction and purification strongly affect protein structure (Fig. 4), the effect of compositional factors on the functional properties of the ingredients can be only deduced by comparing ingredients obtained by comparable processes. For this reason, limited comparable data were available in the literature. In the case of solubility, comparable data were only relevant to purified protein obtained *via* alkaline extraction followed by isoelectric precipitation (AE + IP), since this is the most frequently reported process in the literature (Fig. 2A and B). Solubility data were grouped based on the pH of the aqueous media used for protein ingredient solubilization (Fig. 5). In this regard, although most studies analyze the solubility in a wide pH range (from 2 to 12), only data in the pH range 2–7 were considered, since they are representative of the pH conditions commonly found in food and beverages.

Independently from the protein source, plant proteins presented minimum solubility in the pH range 4–5, which corresponds to the isoelectric region. At the isoelectric pH (pI), the protein surface charge is neutral, which promotes protein-protein association driven by van der Waals, hydrophobic, and hydrogen bonding. By contrast, at pH values far from the pI, the increase in surface protein charge promotes structure stretching and electrostatic repulsion among protein molecules (Ma et al., 2022), thus favoring protein solubility (Fig. 5).

At pH values higher and lower than the pI, proteins from legumes and shell fruit were found to present the highest mean solubility, with values at acidic and neutral pH around 40 and 70%, respectively. Proteins from cereals, pseudocereals, and oilseeds presented comparable solubility values, with a mean value at pH 2–3 and 6–7 around 30 and 50%, respectively. Finally, leaf RuBisCo showed intermediate solubility data at both acid and neutral pH (Fig. 5).

These findings are in agreement with the work of Xu et al. (2023), which is to our knowledge, the only paper comparing the solubility of proteins extracted from different sources using the same protocol. In fact, these Authors showed that proteins obtained from mung bean, adzuki bean, pea, lentil, and soy presented the highest solubility, followed by pseudocereal such as buckwheat, and cereal ones (wheat, oat, and rye).

Taking into consideration the protein profile reported in Table 2, sources rich in water-soluble albumins such as pseudocereals, legumes, and shell fruit are expected to lead to highly soluble protein ingredients. Reversely, sources rich in prolamins and glutelins, which are respectively soluble in dilute alcohol and acid solutions, such as cereals (Table 2) should lead to poorly soluble ingredients.

Nevertheless, the solubility data shown in Fig. 5 does not confirm this hypothesis. For instance, pseudocereal-derived purified protein extracts were found to present relatively low solubility data. This is due to the selectivity of the considered production process: in fact, AE + IP protocols reported in the literature apply pH values for the IP around 4.5, which causes the precipitation of globulins, glutelins and prolamins (Ju et al., 2001; de Sousa et al., 2016). By contrast, at this pH, albumins show a lower propensity towards precipitation, due to their lower size and high-water solubility (Yang et al., 2021). As a result, Fig. 5 does not refer to protein ingredients with protein profiles analogous to that of the plant source they are extracted from.

**Fig. 6:**
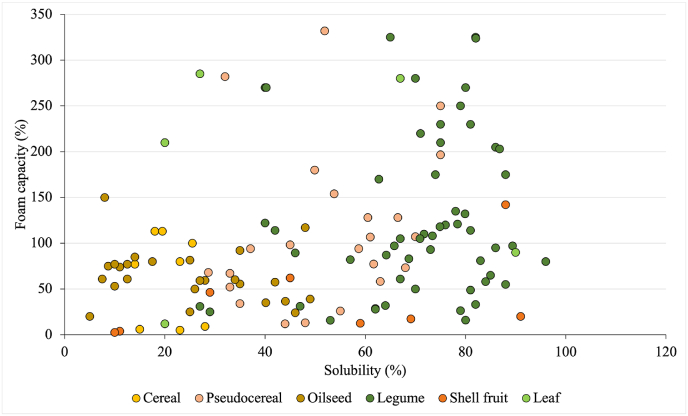
Foaming capacity (FC) as a function of solubility of purified PP ingredients obtained from different protein sources by alkaline extraction followed by isoelectric precipitation. At least 5 FC values were considered for each source. FC is expressed according to equation (2), considering 1% protein solution at pH 6–7. Details regarding the data origin are reported in Supplementary Table S4.

Solubility is the parameter that mostly affects the other technological properties of proteins. In particular, good solubility has been reported to promote the movement of protein to the oil-water or air-water interface, positively affecting both the foam and emulsifying capacity (Ma et al., 2022; Zhang et al., 2021). Based on these considerations, Fig. 6 shows the foam capacity (FC) of plant proteins obtained from different sources as a function of their solubility. Also in this case, to allow for a reliable comparison among the different papers of the database, only purified protein extract obtained using AE + IP were considered; moreover, all the presented FC data were calculated according to equation (2) and using 1% protein solution in the pH range 6–7. Based on the availability of data collected according to these parameters, Fig. 6 compares FC of purified protein extract from pseudocereals, oilseeds, legumes, shell fruit, and leaves. By contrast, not enough data or not comparable data were available in the literature for cereal proteins. Furthermore, indexes relevant to emulsifying capacity, as well as to foam and emulsion stability resulted not comparable due to the high inhomogeneity in methods used for their assessment.

Contrary to what is reported in the literature, Fig. 6 shows no strong dependence of FC on solubility. Rather, a clear effect of plant protein source was found. In fact, despite the high variability, purified protein extract from legumes and pseudocereals showed high FC values, mainly in the range 50–200%. On the opposite, oilseed and shell fruit proteins were associated with low FC values, in the range 50–100 and < 50%, respectively. On the contrary, FC data relevant to leaves proteins could not be grouped, since showing no specific trend. This could be probably associated with the differences in protein structure among the different leaf matrices, as suggested by Famuwagun et al. (2020). Xu et al. (2023) actually suggested that source-related factors other than proteins may play a role in determining protein FC. In particular, specific compounds that are co-extracted during protein ingredient preparation might contribute to its foaming properties. For instance, this is the case of saponins, abundant in legumes and pseudocereals, which present prominent foaming properties (Abugoch et al., 2008; Shrestha et al., 2021).

**Fig. 7:**
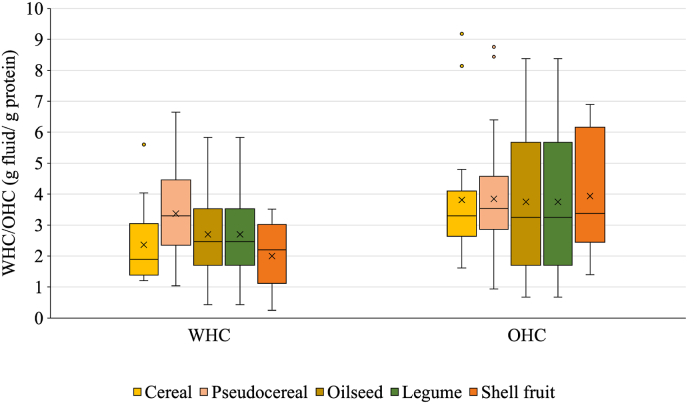
Water (WHC) and oil (OHC) holding capacity of purified PP ingredients obtained from different protein sources by alkaline extraction, followed by isoelectric precipitation. At least 5 values were considered for each source. Details regarding the data origin are reported in Supplementary Table S4.

Fig. 7 reports the WHC and OHC of plant proteins obtained from different plant sources. Compared to the interfacial properties, the simplicity and the higher standardization of the protocols used in the literature to determine these functionalities accounted for a higher number of comparable data.

Independently on the source, WHC values in the range 1–4 g water/g protein ingredient were reported, with pseudocereals and shellfruit showing, respectively the highest and lowest values (Fig. 7). Pseudocereal purified protein ingredients commonly contain co-extracted polysaccharides, which easily entrap large water amounts; on the opposite, the usually high hydrophobicity of oilseed proteins might account for their lower ability to interact with water (Amagliani et al., 2021). The OHC values were higher than the WHC ones, with values in the range of 3.5–4.0 g oil/g protein ingredients, independently from the protein source. Upon mixing with oil, plant protein ingredients do not solubilize in it, and ingredient-oil interaction is mainly driven by physical capillary forces, as well as hydrophobic interactions between the ingredient hydrophobic residues and the oil.

#### 5.1.1 Protein purity

Increased protein purity is usually associated with increased protein solubility (Ogunwolu et al., 2009; Mao and Hua, 2012; Nnamezie et al., 2021; Rayan et al., 2023). Additional components, such as vegetable fibers, polyphenols, and phytic acid, in fact, usually engage in strong interactions with proteins, leading to a reduction of protein ability to interact with water (Karaca et al., 2011a). For example, Krause et al. (2002) reported that the presence of phytic acid and pentosans caused a 50% decrease in the solubility of a flaxseed purified protein ingredient. Similar results were also reported by Rayan et al. (2023), who observed a 10% solubility for purslane protein-rich extract as compared to the 14% solubility of the purified protein extracts obtained from the same plant matrix. The higher solubility of high-purity protein ingredients is also associated with prominent interfacial properties in terms of emulsion and foam formation, as compared to the ones of less refined ingredients. Higher solubility generally promotes protein migration at the interface due to the absence of other compounds that could obstacle this process. However, some studies reported a higher protein solubility for protein-rich extracts as compared to purified ones. For instance, Coelho and Salas-Mellado (2018) showed that chia protein-rich flour had a higher solubility as compared to the purified protein extract. This was attributed to the application of intense purification conditions, which led to denaturation of proteins, compromising their solubility (Potin et al., 2022; Rodsamran and Sothornvit, 2018). Moreover, in some cases, significant amounts of interface-active compounds may occur in low-refined ingredients, resulting in better interfacial properties. This is the case of chia polysaccharides (Lin et al., 1994; Coelho and Salas-Mellado, 2018), which promote higher interfacial properties as compared to those of highly-purified chia proteins. The presence of non-protein compounds could also favor the stability of foams and emulsions, contributing to the increase in viscosity of the continuous phase and the electrostatic repulsion among the dispersed droplets or bubbles (Coelho and Salas-Mellado, 2018; Nnamezie et al., 2021).

According to the literature paper analysis, purified protein extracts usually present WHC and OHC values higher than those of protein flours and protein-rich extracts, despite their higher water solubility and lower amount of fiber, which highly contributes to solvent loading. This was reported by Ogunwolu et al. (2009), Lqari et al. (2002), and Nnamezie et al. (2021) with reference to protein ingredients from cashews, lupins, and okra, respectively. Also in this case, these results can be attributed to the intense protein denaturation induced by the purification process (Fig. 4), which may lead to the expose a high number of hydrophilic residues able to strongly bind water in the unsoluble protein fraction. Flours are most commonly obtained by conventional oven-drying (OD), leading to collapsed powders, presenting thus a limited liquid loading capacity. By contrast, spray- (SD) or freeze-drying (FD) are more commonly used for the production of purified protein extract, leading to highly aerated structures, absorbing huge solvent amounts. Different solvent loading capacities could be thus attributed to the different porosity of the powders rather than to their composition as reported by Manzocco et al. (2024).

Overall, even if composition factors impact the functional properties of plant protein ingredients, the previous discussion underlines how their effect is strongly influenced by the production process, which determines the presence of non-protein compounds, protein denaturation and the level of powder porosity.

Table 6 summarizes the effect of these characteristics on the functional properties of plant protein ingredients.

**Table 6:** Positive (+), negative (−), and null (•) effect of the presence of non-protein compounds, protein denaturation, and powder porosity on the functional properties of plant protein ingredients.

	Presence of non-protein compounds	Protein denaturation	Porosity
Solubility	**−**	**−**	**•**
Interfacial properties	**+ −**	**+ −**	**•**
Solvent holding capacity	**+**	**+**	**+**

For this reason, in the following paragraph, a detailed discussion of the effect of processing factors on protein functionalities is reported.

### 5.2 Effect of processing factors on protein technological functionalities

#### 5.2.1 Extraction and purification technology

As already mentioned, the most commonly applied technology used for the production of purified protein ingredient is AE + IP (Fig. 2). The pH used during the AE phase significantly affects protein functional properties as shown in Table 7.

**Table 7:** Effect of increasing alkaline extraction pH on the functional properties of different plant matrixes and related phenomena.

Functional property		Plant matrix	Mechanisms	Literature
Solubility	Decrease	Quinoa	Denaturation of protein matrix, formation of high-dimension aggregate	Abugoch et al. (2008)
	Decrease	Quinoa	Denaturation, increase in hydrophobicity, aggregation, reduction of surface charge, extraction of low-soluble peptides	Liu et al. (2023)
	Decrease	Quinoa	Denaturation	Ruiz et al. (2016)
	Decrease	Pea	Protein aggregation	Gao et al. (2020)
	Decrease	Amaranth	Increase in surface hydrophobicity, extraction of low-soluble peptides	Das et al. (2021)
	Decrease	Chia	Protein denaturation and aggregation	López et al. (2018)
	Increase	Hemp	Higher purity, change in protein structure, protein-protein interaction, extraction phenolic compounds	Potin et al. (2022)
Interfacial	Increase	Hemp	Higher flexibility, higher hydrophobicity, co-extraction of non-protein compounds	Potin et al. (2022)
	Decrease	Amaranth	Aggregation	Das et al. (2021)
	Decrease	Chia	Protein denaturation and aggregation	López et al. (2018)
Solvent holding capacity	No differences	Quinoa		Abugoch et al. (2008)
	Decrease	Amaranth	Aggregation	Das et al. (2021)
	Increase in WHC Decrease in OHC	Chia	Extraction of non-protein compounds, protein denaturation, exposure of hydrophilic residues, reduction of surface hydrophobicity	López et al. (2018)

Table 7 shows the effect of increasing alkaline extraction pH on the functional properties of different plant matrixes. Different studies reported that the higher the extraction pH, the lower the solubility of the final purified protein extract, due to protein tertiary structure denaturation, leading to protein aggregation. Furthermore, high pH values lead to the ionization and consequent extraction of low-water soluble peptides along with proteins (Das et al., 2021; Liu et al., 2023). Moreover, these conditions favor the extraction of phenolic compounds, which interact with proteins *via* strong covalent bonds in the formation of high-dimension insoluble complexes (Potin et al., 2022). The lower solubility and high protein aggregation induced by high extraction pH also decrease the emulsion and foaming properties of the protein ingredients (Table 7), due to the lower mobility and flexibility of proteins, and the reduced availability of hydrophobic and charged surface groups. In this regard, Das et al. (2021) demonstrated that the increase of the AE pH from 8 to 12 accounted for a decrease in solubility and interfacial properties of an amaranth purified protein extract. Nevertheless, the ability of high AE pH to extract non-protein insoluble compounds, such as fibers, might also account for opposite stability trends (López et al., 2018; Potin et al., 2022). In fact, fibers are well known to inhibit phase separation, based on their ability to control the rheology of the aqueous continuous phase and to jam at the interfaces, exerting a Pickering effect. On the contrary, in the work of Das et al. (2021), an opposite behavior was observed for Amaranth protein extracts. In this case, the protein denaturation effect at high pH probably prevailed over the extraction of polysaccharides, leading to aggregate structures with limited ability to interact with fluids.

As previously described, multiple extraction and purification technologies can be combined, as alternatives to AE + IP (Fig. 2A and B). Table 8 compares the effect of these processes on the functionalities of purified plant protein extract, with those induced by AE + IP, considered as control. The comparison took into consideration data relevant to functional properties assessed in the most frequently applied pH range (6–7). The literature data used for the construction of Table 8 are reported in the supplementary material (Tables S5–S10).

**Table 8:** Effect of different combinations of extraction and purification technology on the techno-functionalities of plant purified protein extract. AE + IP = Alkaline extraction followed by isoelectric precipitation, AE + UF = Alkaline extraction followed by ultrafiltration, SE + D = Salt extraction followed by dialysis, SE + MP = Salt extraction followed by micellar precipitation, SE + IP = Salt extraction followed by isoelectric precipitation. Relevant data are reported in Supplementary Tables S5-S10.

Functional property	Technology				
	AE + IP (control)	AE + UF	SE + D	SE + MP	SE + IP
Solubility	**•**	**•**	**+**	**−**	**−**
FC	**•**	**•**	**+**	**−**	**−**
FS	**•**	**•**	**−**	**−**	**−**
EA/EAI	**•**	**•**	**+**	**−**	**−**
ES/ESI	**•**	**•**	**−**	**−**	**−**
WHC	**•**	**•**	**−**	**+**	**•**
OHC	**•**	**+**	**+**	**•**	**+**

**•:** comparable effect on the functional property.

+: increase in the functional property.

-: decrease in the functional property.

AE + UF usually results in purified protein extract with functional properties similar to those of purified protein extract obtained from AE + IP. For example, pea, chickpea, and lentil purified protein extracts produced from different cultivars by AE + UF and AE + IP, showed comparable foaming and emulsifying properties (Boye et al., 2010a). This suggests that AE is probably the critical step affecting these functionalities since it results in ingredients rich in globulins (Tanger et al., 2020; Zhao et al., 2023), independently of subsequent purification steps.

By contrast, protein-rich ingredients obtained exploiting SE + D were found to present higher protein solubility, and consequently higher foam and emulsion ability (FC and EC/EAI indexes) as compared to the ones obtained from AE + IP (Table 8). SE + D favors not only the extraction of globulins, which are selectively extracted during AE + IP, but also the recovery of smaller, more water soluble and flexible albumins (Karaca et al., 2011b; Stone et al., 2015; Fang et al., 2023). Moreover, SE + D better preserve protein native structure (Fig. 4), thus reducing protein aggregation (Boyle et al., 2018; Fang et al., 2023; Stone et al., 2015), and favoring protein mobility and flexibility (as indicated by the low amount of rigid β structures) (Fang et al., 2023). SE + D also avoids the formation of insoluble complexes between proteins and phytic acid and allows the removal of salts that negatively affect emulsifying and foaming properties (Cheung et al., 2014; R. Zhang et al., 2024). However, SE + D was found to negatively affect emulsion and foaming stability as compared to AE + IP (FS and ES/ESI indexes, Table 8), probably due to the lower polysaccharide content, which in the case of AE + IP increases the viscosity of the continuous phase, reducing the mobility of dispersed air bubbles or oil droplets stabilizing the foam and emulsion (Koysuren et al., 2021). Despite this evidence, Karaca et al. (2011b) reported lower solubility and interfacial properties of purified protein extract from chickpeas, faba beans, lentils, and peas obtained using SE + D as compared to those obtained from AE + IP. It is important to point out that, in this study, SE was associated with the use of buffers which may have caused intense protein denaturation, accounting for the observed lower functionalities. Purified protein extracts obtained by SE + D present lower WHC compared to those obtained using AE + IP, in agreement with their higher solubility. On the opposite, SE + D produces ingredients with higher OHC compared to the AE + IP ones (Stone et al., 2015), possibly due to the lower aggregation of the unsoluble fraction, promoting its interaction with oil.

When SE was followed by micellization (SE + MP), a reduction in plant protein solubility was found as compared to the other technologies (Table 8). MP may induce severe protein denaturation as a consequence of the applied ionic strength. Furthermore, MP, similarly to IP, is selective for globulins, leading to a relatively lower concentration of highly-soluble albumins (Tanger et al., 2020). An even more pronounced negative effect on protein solubility was found by R. Zhang et al. (2024) when studying the solubility of rapeseed proteins obtained by SE + IP. In this case, completely insoluble purified protein extracts were obtained. These effects are probably due to intense protein denaturation and aggregation occurring during MP and IP phases (Koysuren et al., 2021; Zhang et al., 2021), which also accounts for the lower interfacial properties (Table 8). As a result, the resulting purified protein extract usually presents high WHC and OHC (Table 8).

#### 5.2.2 Drying technology

A reduced number of authors have investigated the effect of the drying technology on the functionalities of plant protein ingredients. The comparison is commonly made using FD as control.

OD and VD promote severe denaturation of proteins and the formation of aggregate complexes, with a highly hydrophobic surface layer, negatively affecting solubility. Although these effects should impair foam and emulsion formation, both Dabbour et al. (2022) and Shen et al. (2021) reported that VD proteins from sunflower and amaranth showed increased foam and emulsion capacity, respectively, as compared to the corresponding FD ones, probably due to the instauration of Pickering mechanisms. The strongly collapsed structure of VD and OD purified protein extracts also presented reduced OHC and WHC compared with both SD and FD ones, as demonstrated for both quinoa purified protein extract (Shen et al., 2021) and pea flours (Manzocco et al., 2024).

Compared to FD, SD seems to increase protein solubility, thanks to the lower protein denaturation (Timilsena et al., 2016) and the reduced dimension of the produced particles (Hu et al., 2009; Shen et al., 2021). Accordingly, higher interfacial properties were registered for soy and chia purified protein extracts submitted to SD compared to FD ones (Hu et al., 2009; Timilsena et al., 2016). SD ingredients usually show lower WHC and OHC, due to their higher solubility and lower porosity (Özdemir et al., 2022). Nevertheless, an opposite effect of FD and SD was found in the work of Shen et al. (2021) on quinoa purified protein extract, highlighting the need for further studying the effect of drying technology on the functionalities of plant protein ingredients. In this regard, the processing conditions applied during SD could significantly impact the final protein functionality. In the work of Burger et al. (2022), the dimension of the spray drier (lab and pilot scale) was found to strongly affect solubility and the other functional properties. Similarly, the feed rate, atomization speed, inlet and outlet temperature, airflow, and residence time applied during SD can significantly affect the properties of plant proteins, as reported in the review of Muhoza et al. (2024).

## 6 Conclusions

In the last decades, the interest of both the scientific community and food industries for plant protein ingredients able to substitute animal ones in food formulation has grown exponentially. Despite a huge number of papers investigated the physicochemical as well as the functional properties of proteins deriving from different plant sources, fine orienteering among this piece of science is particularly challenging duo to

- absence of a non-ambiguous classification of plant protein ingredients accounting for both protein concentration and production process;
- lack of standardized and shared protocols for the evaluation of the functional properties of protein ingredients;
- partial characterization of developed ingredients considering only the physicochemical or the functional properties;
- complex interplay among compositional and processing factors on protein structure and thus on their functionality;
- occurrence in plant protein ingredients of non-protein compounds with difficulty predictable effect on functionality.

In the light of this intricated scenario, answering the question “What is the process to be applied to a given plant matrix in order to obtain a protein ingredient with the desired functional properties?” is an arduous task.

The critical literature analysis carried out in this review, based on the comparison of comparable scientific evidence, highlighted that the plant source affects plant protein ingredient functionalities, due to the inherent differences in the composition of protein fractions according to the botanical origin. In particular, legume proteins, which are mainly represented by globulins, show the highest solubility and interfacial properties, while cereal ones, rich in prolamins and glutenins, showed the poorest functional properties. Moreover, ingredients characterized by a high purity generally show higher solubility, interfacial properties, and water and oil holding capacity. However, the presence of components other than proteins could significantly contribute to the interfacial properties of the ingredient, through the formation and stabilization of interfaces between water and air or oil.

Besides composition, the knowledge of the technological history is required to select an ingredient with the desired functionalities, since different extraction, purification and drying methods significantly affect the functional properties of plant proteins. Alkaline extraction, isoelectric precipitation and freeze-drying is the process mostly used in academic research to obtain PP ingredients. However, this process is not used by food companies engaged in the preparation of PP ingredients, leading to incomparable results. Compared to alkaline extraction, isoelectric precipitation and freeze-drying, salt extraction and physical purification methods, such as dialysis and ultrafiltration, followed by spray drying reduce protein denaturation, resulting in increased solubility and interfacial properties.

The analysis of the available literature evidenced the lack of a systematic approach in the field of plant protein research. An important step ahead could be represented by the establishment of a widely recognized method of ingredient preparation, which could be used as a reference to allow comparison with other methodologies. A valuable reference candidate could be alkaline extraction followed by isoelectric precipitation performed at precisely defined environmental and processing conditions. Furthermore, the concomitant evaluation with harmonized methods and procedures of the physico-chemical and the functional properties of plant protein ingredients is needed to untangle the complex relationship among these properties and how they are affected by composition, process, and source.

This approach is required to turn the wide, detailed but not comparable scientific literature currently available into user-friendly information able to

i. assist researchers in investigating the plant proteins and how their properties are affected by different composition and processing factors;
ii. answer the impelling industrial demand for decision tools, supporting the choice of food company operators for plant protein ingredients with optimized functionalities.

## CRediT authorship contribution statement

**Lorenzo Barozzi:** Conceptualization, Data curation, Formal analysis, Investigation, Methodology, Writing – original draft, Writing – review & editing. **Stella Plazzotta:** Conceptualization, Methodology, Supervision, Writing – original draft, Writing – review & editing. **Ada Nucci:** Writing – review & editing. **Lara Manzocco:** Conceptualization, Supervision, Project administration, Funding acquisition, Writing – review & editing.

## Funding

This research was funded by 10.13039/501100000780European Union FSE REACT-EU, 10.13039/100023500PON Ricerca Innovazione 2014–2020.

This work was financed by the EU- NextGenerationEU Project “Upcycling pea waste side streams for developing future food ingredients -UPea"; PRIN Bando 2022; Prot. 20222P5C3E.

**Figure:**
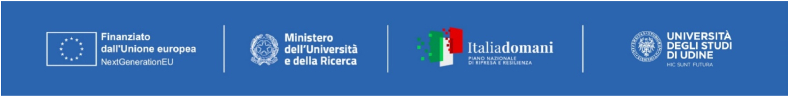

## Declaration of competing interest

The authors declare that they have no known competing financial interests or personal relationships that could have appeared to influence the work reported in this paper.

## Data Availability

Data will be made available on request.
