# Supplementary material for: Elucidating the role of compositional and processing variables in tailoring the technological functionalities of plant protein ingredients
Source: Curr Res Food Sci. 2025 Jan 9;10:100971. doi: 10.1016/j.crfs.2025.100971 (PMC11795097; doi:10.1016/j.crfs.2025.100971)
Supplement: Multimedia component 1 [file mmc1.docx]

**Supplementary Tables:**

Table S1. Combinations of the search words used for the article research.

| Combination number | Search word combination |
| --- | --- |
| 1 | plant AND protein AND technological AND properties |
| 2 | plant AND protein AND functional AND properties |
| 3 | plant AND protein AND extraction |
| 4 | plant AND protein AND alkaline AND extraction |
| 5 | plant AND protein AND salt AND extraction |
| 6 | plant AND protein AND micellar AND precipitation |
| 7 | cereal AND protein AND technological AND properties |
| 8 | pseudocereal AND protein AND technological AND properties |
| 9 | legume AND protein AND technological AND properties |
| 10 | oilseed AND protein AND technological AND properties |
| 11 | leaves AND protein AND technological AND properties |
| 12 | cereal AND protein AND functional AND properties |
| 13 | pseudocereal AND protein AND functional AND properties |
| 14 | legume AND protein AND functional AND properties |
| 15 | oilseed AND protein AND functional AND properties |
| 16 | leaves AND protein AND functional AND properties |
| 17 | cereal AND protein AND extraction |
| 18 | pseudocereal AND protein AND extraction |
| 19 | legume AND protein AND extraction |
| 20 | oilseed AND protein AND extraction |
| 21 | leaves AND protein AND extraction |
| 22 | plant AND protein AND isolate |
| 23 | protein AND isolate AND functional AND properties |
| 24 | plant AND protein AND physicochemical AND properties |
| 25 | protein AND concentrate AND functional AND properties |
| 26 | protein AND physicochemical AND functional AND properties |
| 27 | protein AND emulsifying AND properties |
| 28 | plant AND protein AND drying |
| 29 | plant AND protein AND solubility |

Table S2. List of plant matrices used as search words during the first screening of the keywords.

| Word number | Search word | Word number | Search word | Word number | Search word |
| --- | --- | --- | --- | --- | --- |
| 1 | Alfaalfa | 16 | Fava | 31 | Peony |
| 2 | Almond | 17 | Flaxssed | 32 | Pepper |
| 3 | Amaranth | 18 | Hemp | 33 | Primrose |
| 4 | Avocado | 19 | Huauzontle | 34 | Pseudocereal |
| 5 | Barley | 20 | Jackfruit | 35 | Quinoa |
| 6 | Bean | 21 | Leaf | 36 | Rapeseed |
| 7 | Buckwheat | 22 | Leaf | 37 | Rice |
| 8 | Camelina | 23 | Legume | 38 | Rye |
| 9 | Cashew | 24 | Lentil | 39 | Sesame |
| 10 | Cereal | 25 | Lime | 40 | Shellfruit |
| 11 | Chia | 26 | Lupine | 41 | Soy |
| 12 | Chickpea | 27 | Oat | 42 | Sunflower |
| 13 | Coconut | 28 | Oilseed | 43 | Tamarind |
| 14 | Cotton | 29 | Okra | 44 | Wheat |
| 15 | Faba | 30 | Pea |  |  |

Table S3. Example of non-considered article title and reason.

| Example number | Title | Exclusion reason |
| --- | --- | --- |
| 1 | Improved functional properties of pasta: Enrichment with amaranth seed flour and dried amaranth leaves | Development of specific food product |
| 2 | Physicochemical and functional properties of Thai organic rice flour | Functional properties of flour, not of extracted proteins |
| 3 | Pea pod, broad bean pod and okara, potential sources of functional compounds | Not specific for protein |
| 4 | Mechanistic study on the nanocomplexation between curcumin and protein hydrolysates from Great Northern bean (Phaseolus vulgaris L.) for delivery applications in functional foods | Complexation with other compound and delivery study |
| 5 | Improved thermal and oxidation stabilities of pickering high internal phase emulsions stabilized using glycated pea protein isolate with glycation extent | Glycosilation of protein |
| 6 | Effects of NaCl concentration and temperature on fibrillation, structure, and functional properties of soy protein isolate fibril dispersions | Study of fibrillation |
| 7 | Influence of low molecular weight surfactants on the stability of model infant formula emulsions based on hydrolyzed rice protein | Effect of surfactant |
| 8 | Effect of Solar Drying Methods on Proximate Composition, Sugar Profile and Organic Acids of Mango Varieties in Tanzania | Not relevant to protein |
| 9 | Modelling the effect of prebiotics, probiotics and other functional additives on the growth, feed intake and feed conversion of European sea bass (Dicentrarchus labrax) juveniles | Not relevant to protein |
| 10 |  |  |

Table S4. Relevant literature exploited in the development of the different figures, arranged in alphabetic order.

| Figure number | Relevant literature |
| --- | --- |
| 2 | Abugoch et al., 2008, 2010; Achouri et al., 2012, 2020; Acquah et al., 2020; Agboola et al., 2005; Ahlström et al., 2022; Alavi et al., 2021; Albe-Slabi et al., 2022; Alonso-Miravalles et al., 2019; Alu’datt et al., 2012; Aluko et al., 2001; Alzuwaid et al., 2020; Amirshaghaghi et al., 2017; Aryee & Boye, 2017; Avramenko et al., 2013; Bao et al., 2022; M. Barac et al., 2010, 2012; M. B. Barac et al., 2015; Benzitoune et al., 2022; Bilgi & Çelik, 2004; Biswas & Sit, 2020; Bocarando-Guzmán et al., 2022; Bolontrade et al., 2016; J. I. Boye et al., 2010; Boyle et al., 2018; Brishti et al., 2020; Bruce et al., 2019; Brückner-Gühmann et al., 2018; Bučko et al., 2016; Burger et al., 2022; Calderón-Chiu et al., 2021; Cattan et al., 2022; Chandi & Sogi, 2007; L. Chang et al., 2022; Cheng et al., 2022; Cheung et al., 2014; Chew, 2003; Choe et al., 2022; Coelho & Salas-Mellado, 2018; Condés et al., 2009; Cordero-de-los-Santos et al., 2005; Cui et al., 2020; Dabbour et al., 2018, 2022; Dai et al., 2023; Dapčević-Hadnađev et al., 2018, 2019; Das et al., 2021; García Arteaga et al., 2021; T. S. P. de Souza et al., 2020; Domínguez et al., 2023; Dong et al., 2011; El-Adawy et al., 2001; Esmaeili et al., 2016; Evangelho et al., 2017; Famuwagun et al., 2020; Fang et al., 2023; Fathollahy et al., 2021; Feyzi et al., 2018; Figueroa-González et al., 2022; Fu et al., 2022; L. L. Gao et al., 2018; García Arteaga et al., 2020; J. Ge et al., 2021, 2022; Y. Ge et al., 2000; Geerts et al., 2017; Ghodsvali et al., 2005; Ghribi et al., 2015; Gong et al., 2016; González-Pérez et al., 2005; Guan et al., 2007; Hadidi et al., 2021; Hadnađev et al., 2018; Hansen et al., 2022; Hayati Zeidanloo et al., 2019; Hojilla-Evangelista et al., 2017; Hojilla‐Evangelista et al., 2018; Houde et al., 2018; Iyenagbe et al., 2017; Jia et al., 2021; M. Joshi et al., 2011; Julakanti et al., 2023; Kang et al., 2022; Karabulut & Yemiş, 2022; Karaca et al., 2011a, 2011b; Kaushik et al., 2016; Konak et al., 2014; Ladjal-Ettoumi et al., 2016; Lam et al., 2017; Lawal, 2004; Lee et al., 2021; L’hocine et al., 2006; L. Li et al., 2020; M. Li et al., 2018; R. Li & Xiong, 2021; X. Li et al., 2023; Liang & Tang, 2013; M. Ma et al., 2018; C. Li et al., 2020; Y. Lin et al., 2021; Lindemann et al., 2022; C. Liu et al., 2018; F. Liu et al., 2013; F.-F. Liu et al., 2022; X. Liu et al., 2022; Lopes Lessa et al., 2022; López-Castejón et al., 2020; López-Monterrubio et al., 2020; Lqari et al., 2002; M. Ma et al., 2018; Y. Ma et al., 2023; Mahdavian Mehr & Koocheki, 2020; Malik et al., 2017; Malik & Saini, 2017, 2018; Malomo et al., 2014; Manamperi et al., 2011; X. Mao & Hua, 2012; X.-Y. Mao & Hua, 2014; Martin et al., 2019; Martínez‐Flores et al., 2006; Martínez-Velasco et al., 2018a; Martínez-Velasco et al., 2018b; Meenmanee et al., 2022; Mel et al., 2023; Mir et al., 2021; Mohamed et al., 2007; Mohan & Mellem, 2020; Mokni Ghribi et al., 2015; Molina et al., 2001; Morales et al., 2015; Mozafarpour et al., 2022; Mun et al., 2016; Nazari et al., 2018; Neto et al., 2001; Ngo & Shahidi, 2021; Nieuwland et al., 2021; Nnamezie et al., 2021; Ochoa-Rivas et al., 2017; Omura et al., 2021; Opazo-Navarrete et al., 2022; Özdemir et al., 2022; Papalamprou et al., 2010; Paraman, Hettiarachchy, & Schaefer, 2007; Paraman, Hettiarachchy, Schaefer, et al., 2007; Pastor-Cavada et al., 2010; Peyrano et al., 2016; Pinciroli et al., 2009; Piornos et al., 2015; Potin et al., 2022; Premkumar et al., 2022; Prosekov et al., 2018; Qadir & Wani, 2023; X. Qin et al., 2022; Z. Qin et al., 2013; Rajchman et al., 2022; Ramani et al., 2021; Rayan et al., 2023; Rodríguez-Ambriz et al., 2005; Rodsamran & Sothornvit, 2018; Ruckmangathan et al., 2022; Ruiz et al., 2016; Saetae et al., 2011; Salazar‐Vega et al., 2021; Salcedo-Chávez et al., 2002; Salgado et al., 2011; Samsalee & Sothornvit, 2021; Sánchez-Reséndiz et al., 2019; Saraiva et al., 2021; Saricaoglu et al., 2018; Schlegel et al., 2020; Sha & Xiong, 2022; Sharma et al., 2016; P. Shen et al., 2020; Y. Shen et al., 2021; Shevkani et al., 2014; Shevkani, Kaur, et al., 2015; Shevkani, Singh, et al., 2015; Shi et al., 2020; Shi & Nickerson, 2022; Singh et al., 2021; Steffolani et al., 2016; Stone, Avarmenko, et al., 2015; Stone, Karalash, et al., 2015; Subaşı et al., 2020; Sun et al., 2021; Sze-Tao & Sathe, 2000; Taherian et al., 2011; E.-S. Tan et al., 2014; C.-H. Tang et al., 2006, 2009; S. Tang et al., 2003; X. Tang et al., 2021; Tao et al., 2019; Tatar et al., 2015; Teh et al., 2014; Thaiphanit & Anprung, 2016; Timilsena et al., 2016; Toews & Wang, 2013; Tomotake et al., 2002; Ulloa et al., 2017; Ventureira et al., 2012; Vioque et al., 2012; Vogelsang-O’Dwyer et al., 2020, 2023; J.-S. Wang et al., 2019; L. Wang et al., 2021, 2023; X.-S. Wang et al., 2008; A. A. Wani et al., 2011; I. A. Wani et al., 2015; Withana-Gamage et al., 2011; H. Wu et al., 2009; J. Wu et al., 2022; L. Wu et al., 2021; Xiong et al., 2018; P.-W. Xu et al., 2023; X. Xu et al., 2023; Y. Xu et al., 2017; Yan et al., 2020; Yancheshmeh et al., 2022; J. Yang, de Wit, et al., 2022; J. Yang et al., 2021; J. Yang, Mocking-Bode, et al., 2022; K. Yang et al., 2021; Yao et al., 2023; Yin et al., 2010; Yoshie-Stark et al., 2008; Youshanlouei et al., 2022; Yu et al., 2017; Yue et al., 2021; Y. Zhang et al., 2023; G. Zhao et al., 2011; Q. Zhao, Wang, et al., 2021; Q. Zhao, Yan, et al., 2021; Y. Zhao et al., 2021; Y. Zhao, Yuan, et al., 2023; Zheng et al., 2020; Zhu et al., 2018. |
| 3 | Das et al., 2021; Lqari et al., 2002; Peyrano et al., 2016; Ruiz et al., 2016 |
| 5 | Abugoch et al., 2008, 2010; Achouri et al., 2012, 2020; Agboola et al., 2005; Alzuwaid et al., 2020; Amirshaghaghi et al., 2017; Aryee & Boye, 2017; Bao et al., 2022; M. Barac et al., 2010; M. B. Barac et al., 2015; Bilgi & Çelik, 2004; Biswas & Sit, 2020; Bocarando-Guzmán et al., 2022; J. I. Boye et al., 2010; Brishti et al., 2020; Bruce et al., 2019; Bučko et al., 2016; L. Chang et al., 2022; Chew, 2003; Choe et al., 2022; Cordero-de-los-Santos et al., 2005; Dabbour et al., 2018, 2022; Das et al., 2021; T. S. P. de Souza et al., 2020; Dong et al., 2011; Famuwagun et al., 2020; Fang et al., 2023; Fathollahy et al., 2021; Figueroa-González et al., 2022; L. L. Gao et al., 2018; J. Ge et al., 2021, 2022; Y. Ge et al., 2000; Ghribi et al., 2015; Hadidi et al., 2021; Hadnađev et al., 2018; Hayati Zeidanloo et al., 2019; Hojilla-Evangelista et al., 2017; Julakanti et al., 2023; Lawal, 2004; L. Li et al., 2020; M. Li et al., 2018; Liang & Tang, 2013; M. Ma et al., 2018; C. Li et al., 2020; Y. Lin et al., 2021; C. Liu et al., 2018; F.-F. Liu et al., 2022; López-Monterrubio et al., 2020; M. Ma et al., 2018; Malik et al., 2017; Malik & Saini, 2017; Malomo et al., 2014; X. Mao & Hua, 2012; Martin et al., 2019; Meenmanee et al., 2022; Mohan & Mellem, 2020; Mun et al., 2016; Neto et al., 2001; Ngo & Shahidi, 2021; Nieuwland et al., 2021; Nnamezie et al., 2021; Piornos et al., 2015; Potin et al., 2022; Premkumar et al., 2022; Prosekov et al., 2018; Qadir & Wani, 2023; Rodríguez-Ambriz et al., 2005; Rodsamran & Sothornvit, 2018; Ruckmangathan et al., 2022; Ruiz et al., 2016; Saetae et al., 2011; Salazar‐Vega et al., 2021; Salcedo-Chávez et al., 2002; Y. Shen et al., 2021; Shevkani et al., 2014; Shevkani, Kaur, et al., 2015; Shevkani, Singh, et al., 2015; Shi et al., 2020; Steffolani et al., 2016; S. Tang et al., 2003; Timilsena et al., 2016; Tomotake et al., 2002; J.-S. Wang et al., 2019; L. Wang et al., 2021; I. A. Wani et al., 2015; Withana-Gamage et al., 2011; X. Xu et al., 2023; Yan et al., 2020; J. Yang et al., 2021; K. Yang et al., 2021; Yoshie-Stark et al., 2008; Youshanlouei et al., 2022; Y. Zhang et al., 2023; G. Zhao et al., 2011. |
| 6 | Achouri et al., 2020; Bao et al., 2022; M. Barac et al., 2010; M. B. Barac et al., 2015; Biswas & Sit, 2020; Bocarando-Guzmán et al., 2022; Chandi et al., 2007; L. Chang et al., 2022; Cheng et al., 2022; Choe et al., 2022; Cordero-de-los-Santos et al., 2005; Dabbour et al., 2022; Das et al., 2021; Dong et al., 2011; Fang et al., 2023; Fu et al., 2022; L. L. Gao et al., 2018; J. Ge et al., 2021; Hadidi et al., 2021; Kang et al., 2022; C. C. Liu et al., 2018; M. Liu et al., 2023; López-Monterrubio et al., 2020; M. Ma et al., 2018; Malik et al., 2017; Malomo et al., 2014; X. Mao & Hua, 2012; Martin et al., 2019; Meenmanee et al., 2022; Mohan & Mellem, 2020; Ngo & Shahidi, 2021; Ogunwolu et al., 2009; Piornos et al., 2015; Premkumar et al., 2022; Qin et al., 2013; Saetae et al., 2011; Shevkani et al., 2014; Shevkani, Kaur, et al., 2015; Shevkani, Singh, et al., 2015; Shi et al., 2020, 2022; Steffolani et al., 2016; Stone et al., 2022; Timilsena et al., 2016; I. A. Wani et al., 2015 X. Xu et al., 2023; Yan et al., 2020; J. Yang et al., 2021; K. Yang et al., 2021; Yue et al., 2021; Y. Zhang et al., 2023. |
| 7 | Abugoch et al., 2008, 2010; Achouri et al., 2012, 2020; Alzuwaid et al., 2020; Amirshaghaghi et al., 2017; Aryee & Boye, 2017; Benzitoune et al., 2022; Biswas & Sit, 2020; J. I. Boye et al., 2010; Brishti et al., 2020; Chandi et al., 2007; L. Chang et al., 2022; Dabbour et al., 2018, 2022; Das et al., 2021; T. S. P. de Souza et al., 2020; Dong et al., 2011; Fathollahy et al., 2021; L. L. Gao et al., 2018; J. Ge et al., 2021, 2022; Ghribi et al., 2015; Hadidi et al., 2021; Hadnađev et al., 2018; Hayati Zeidanloo et al., 2019; M. Joshi et al., 2011; Julakanti et al., 2023; Lam et al., 2017; Lawal, 2004; L. Li et al., 2020; M. Li et al., 2018; Liang & Tang, 2013; M. Ma et al., 2018; C. Li et al., 2020; Y. Lin et al., 2021; C. Liu et al., 2018; J. Liu et al., 2019; M. Liu 1023; López-Monterrubio et al., 2020; M. Ma et al., 2018; Malik et al., 2017; Malik & Saini, 2017; X. Mao & Hua, 2012; Mir et al., 2021; Mohan & Mellem, 2020; Neto et al., 2001; Ngo & Shahidi, 2021; Nnamezie et al., 2021; Ogunwolu et al., 2009; Piornos et al., 2015; Prosekov et al., 2018; Qadir & Wani, 2023; Rodríguez-Ambriz et al., 2005; Rodsamran & Sothornvit, 2018; Ruckmangathan et al., 2022; Y. Shen et al., 2021; Shevkani et al., 2014; Shevkani, Kaur, et al., 2015; Shevkani, Singh, et al., 2015; Shi et al., 2020, 2022; Steffolani et al., 2016; Stone et al., 2015; Timilsena et al., 2016; Tomotake et al., 2002; J.-S. Wang et al., 2019; L. Wang et al., 2021; I. A. Wani et al., 2015; Withana-Gamage et al., 2011; X. Xu et al., 2023; J. Yang et al., 2021; Youshanlouei et al., 2022; Y. Zhang et al., 2023. |

Table S5. Effect of different combinations of extraction and purification technology on the solubility of plant purified protein extract. AE+IP = Alkaline extraction followed by isoelectric precipitation, AE + UF = Alkaline extraction followed by ultrafiltration, SE + D = Salt extraction followed by dialysis, SE + MP = Salt extraction followed by micellar precipitation, SE + IP = Salt extraction followed by isoelectric precipitation. Solubility is expressed according to equation 1, as the % amount of proteins that is solubilized upon kinetic determination, as compared to the total proteins present in the purified protein extract.

|  |  | Solubility (%) |  |  |  | Reference |
| --- | --- | --- | --- | --- | --- | --- |
|  |  | **Technique** |  |  |  |  |
| **Source** |  | **AE+IP** | **AE+UF** | **SE+D** | **SE+MP** |  |
| Legumes | Pea | 63-64 |  | 86-91 | 43-49 | Stone et al., 2015 |
|  |  | 61 |  | 38 |  | Karaca et al., 2011b |
|  |  | 70 | 56 |  |  | J. I. Boye et al., 2010 |
|  |  | 77 |  | 96 | 25 | Tanger et al., 2020 |
|  | Chickpea | 91 |  | 30 |  | Karaca et al., 2011b |
|  |  | 55 | 33 |  |  | J. I. Boye et al., 2010 |
|  | Faba bean | 90 |  | 53 |  | Karaca et al., 2011b |
|  | Lentil | 91 |  | 90 |  | Karaca et al., 2011b |
|  |  | 58-59 | 62-73 |  |  | J. I. Boye et al., 2010 |
| Oilseeds | Camelina | 16-18 |  | 43-50 |  | Boyle et al., 2018 |
|  | Hemp | 8 | 12 |  |  | Fang et al., 2023 |
| Pseudocereal | Amaranth | 47 |  |  | 10 | Cordero-de-los-Santos et al., 2005 |

Table S6. Effect of different combinations of extraction and purification technology on the foam stability (FS) of plant purified protein extract. AE+IP = Alkaline extraction followed by isoelectric precipitation, AE + UF = Alkaline extraction followed by ultrafiltration, SE + D = Salt extraction followed by dialysis, SE + MP = Salt extraction followed by micellar precipitation, SE + IP = Salt extraction followed by isoelectric precipitation.

|  |  | FS (%) |  |  |  |  | Reference |
| --- | --- | --- | --- | --- | --- | --- | --- |
|  |  | **Technology** |  |  |  |  |  |
| **Source** |  | **AE+IP** | **AE+UF** | **SE+D** | **SE+MP** | **SE+IP** |  |
| Legumes | Pea | 68.0-69.6 |  | 48.9-69.6 | 52.8-77.8 |  | Stone et al., 2015 |
|  |  | 68 | 68 |  |  |  | J. I. Boye et al., 2010 |
|  | Lentil | 79 | 67 |  |  |  | J. I. Boye et al., 2010 |
|  | Chickpea | 86 | 97 |  |  |  | J. I. Boye et al., 2010 |
| Oilseeds | Camelina | 61 |  | 72 |  |  | Boyle et al., 2018 |
|  | Hemp | 85 |  | 83 |  |  | Fang et al., 2023 |
|  | Rapeseed | 26 |  | 73 |  | 4 | R. Zhang et al., 2024 |
| Pseudocereals | Amaranth | 100 |  |  | 68 |  | Cordero-de-los-Santos et al., 2005 |

Table S7. Effect of different combinations of extraction and purification technology on the emulsifying capacity (EC) and emulsifying activity index (EAI) of plant purified protein extract. AE+IP = Alkaline extraction followed by isoelectric precipitation, AE + UF = Alkaline extraction followed by ultrafiltration, SE + D = Salt extraction followed by dialysis, SE + MP = Salt extraction followed by micellar precipitation, SE + IP = Salt extraction followed by isoelectric precipitation.

|  |  | EC/EAI | | | | | Reference |
| --- | --- | --- | --- | --- | --- | --- | --- |
|  |  | **Technology** |  |  |  |  |  |
|  |  | **AE+IP** | **AE+UF** | **SE+D** | **SE+MP** | **SE+IP** |  |
| Legumes | Pea | 187.5-193.7% |  | 193.7-243.7% |  |  | Stone et al., 2015 |
|  |  | 42.87 m^2^/g |  | 42.73 m^2^/g |  |  | Karaca et al., 2011b |
|  |  | 4.7 m^2^/g | 4.6 m^2^/g |  |  |  | J. I. Boye et al., 2010 |
|  | Lentil | 44.51 m^2^/g |  | 37.17 m^2^/g |  |  | Karaca et al., 2011b |
|  |  | 4.8 m^2^/g | 5.0 m^2^/g |  |  |  | J. I. Boye et al., 2010 |
|  | Chickpea | 5.6 m^2^/g | 5.5 m^2^/g |  |  |  | J. I. Boye et al., 2010 |
|  |  | 47.9 m^2^/g |  | 33.83 m^2^/g |  |  | Karaca et al., 2011b |
|  | Faba | 44.29 m^2^/g |  | 37.11 m^2^/g |  |  | Karaca et al., 2011b |
| Oilseeds | Camelina | 180 m^2^/g |  | 520 m^2^/g |  |  | Boyle et al., 2018 |
|  | Hemp | 3.7 m^2^/g |  | 6.2 m^2^/g |  |  | Fang et al., 2023 |
|  | Canola | 15 m^2^/g |  | 45 m^2^/g |  |  | Karaca et al., 2011a |
|  | Flaxseed | 39 m^2^/g |  | 41 m^2^/g |  |  | Karaca et al., 2011a |
|  | Rapessed | 7.3 m^2^/g |  | 5.1 m^2^/g |  | 2.5 m^2^/g | Fang et al., 2023 |
|  | Sesame | 6 m^2^/g |  |  |  | 2 m^2^/g | Koysuren et al., 2021 |
| Pseudocereals | Amaranth | 37% |  |  | 42% |  | Cordero-de-los-Santos et al., 2005 |

Table S8. Effect of different combinations of extraction and purification technology on the emulsifying stability (ES) and emulsifying stability index (ESI) of plant purified protein extract. AE+IP = Alkaline extraction followed by isoelectric precipitation, AE + UF = Alkaline extraction followed by ultrafiltration, SE + D = Salt extraction followed by dialysis, SE + MP = Salt extraction followed by micellar precipitation, SE + IP = Salt extraction followed by isoelectric precipitation.

|  |  | ES/ESI (%) |  |  |  |  | Reference |
| --- | --- | --- | --- | --- | --- | --- | --- |
|  |  | **Technology** |  |  |  |  |  |
|  |  | **AE+IP** | **AE+UF** | **SE+D** | **SE+MP** | **SE+IP** |  |
| Legumes | Pea | 96.7-99.0 % |  | 97.0-99.6 % | 99.5-99.7 % |  | Stone et al., 2015 |
|  |  | 12.4 |  | 10.89 |  |  | Karaca et al., 2011b |
|  |  | 18.2 min | 17.8 |  |  |  | J. I. Boye et al., 2010 |
|  | Lentil | 86.79 |  | 11.02 |  |  | Karaca et al., 2011b |
|  |  | 17.8 | 18.4 |  |  |  | J. I. Boye et al., 2010 |
|  | Chickpea | 82.94 min |  | 10.92 |  |  | Karaca et al., 2011b |
|  |  | 19.5 | 19.5 |  |  |  | J. I. Boye et al., 2010 |
|  | Faba | 69.39 |  | 10.97 |  |  | Karaca et al., 2011b |
| Oilseed | Sesame | 100 min |  |  |  | 50 min | Koysuren et al., 2021 |
|  | Camelina | 16 min |  | 18 min |  |  | Boyle et al., 2018 |
|  |  | 62% |  |  | 73% |  | Cordero-de-los-Santos et al., 2005 |
|  | Hemp | 42 |  | 18.5 |  |  | Fang et al., 2023 |
|  | Canola | 10 min |  | 12 |  |  | Karaca et al., 2011a |
|  | Flaxseed | 12 |  | 16 |  |  | Karaca et al., 2011a |
|  | Rapessed | 86 |  | 13 |  | 34 | R. Zhang et al., 2024 |

Table S9. Effect of different combinations of extraction and purification technology on the water holding capacity (WHC) of plant purified protein extract. AE+IP = Alkaline extraction followed by isoelectric precipitation, AE + UF = Alkaline extraction followed by ultrafiltration, SE + D = Salt extraction followed by dialysis, SE + MP = Salt extraction followed by micellar precipitation, SE + IP = Salt extraction followed by isoelectric precipitation.

|  |  | WHC (g/g) |  |  |  |  | Reference |
| --- | --- | --- | --- | --- | --- | --- | --- |
|  |  | **Technology** |  |  |  |  |  |
|  |  | **AE+IP** | **AE+UF** | **SE+D** | **SE+IP** | **SE+MP** |  |
| Legumes | Pea | 2.4-2.6 |  | 0.3-2.6 |  | 3.2-3.6 | Stone et al., 2015 |
|  |  | 4.4 | 3.8 |  |  |  | J. I. Boye et al., 2010 |
|  | Lentil | 3.9 | 3.5 |  |  |  | J. I. Boye et al., 2010 |
|  | Chickpea | 3.1 | 2.6 |  |  |  | J. I. Boye et al., 2010 |
| Oilseed | Rapessed | 3.0 |  | 3.2 | 3.0 |  | R. Zhang et al., 2024 |

Table S10. Effect of different combinations of extraction and purification technology on the oil holding capacity (OHC) of plant purified protein extract. AE+IP = Alkaline extraction followed by isoelectric precipitation, AE + UF = Alkaline extraction followed by ultrafiltration, SE + D = Salt extraction followed by dialysis, SE + MP = Salt extraction followed by micellar precipitation, SE + IP = Salt extraction followed by isoelectric precipitation.

|  |  | OHC (g/g) |  |  |  |  | Reference |
| --- | --- | --- | --- | --- | --- | --- | --- |
|  |  | **Technology** |  |  |  |  |  |
|  |  | **AE+IP** | **AE+UF** | **SE+D** | **SE+IP** | **SE+MP** |  |
| Legumes | Pea | 3.5-3.8 |  | 5.2-5.4 |  | 3.6 | Stone et al., 2015 |
|  |  | 1.2 | 1.7 |  |  |  | J. I. Boye et al., 2010 |
|  | Lentil | 1.25 | 1.35 |  |  |  | J. I. Boye et al., 2010 |
|  | Chickpea | 1.3 | 1.2 |  |  |  | J. I. Boye et al., 2010 |
| Oilseed | Rapessed | 1.5 |  | 2.5 | 2.3 |  | R. Zhang et al., 2024 |
