# Supplementary figures and images for: Elucidating the role of compositional and processing variables in tailoring the technological functionalities of plant protein ingredients
Source: Curr Res Food Sci. 2025 Jan 9;10:100971. doi: 10.1016/j.crfs.2025.100971 (PMC11795097; doi:10.1016/j.crfs.2025.100971)

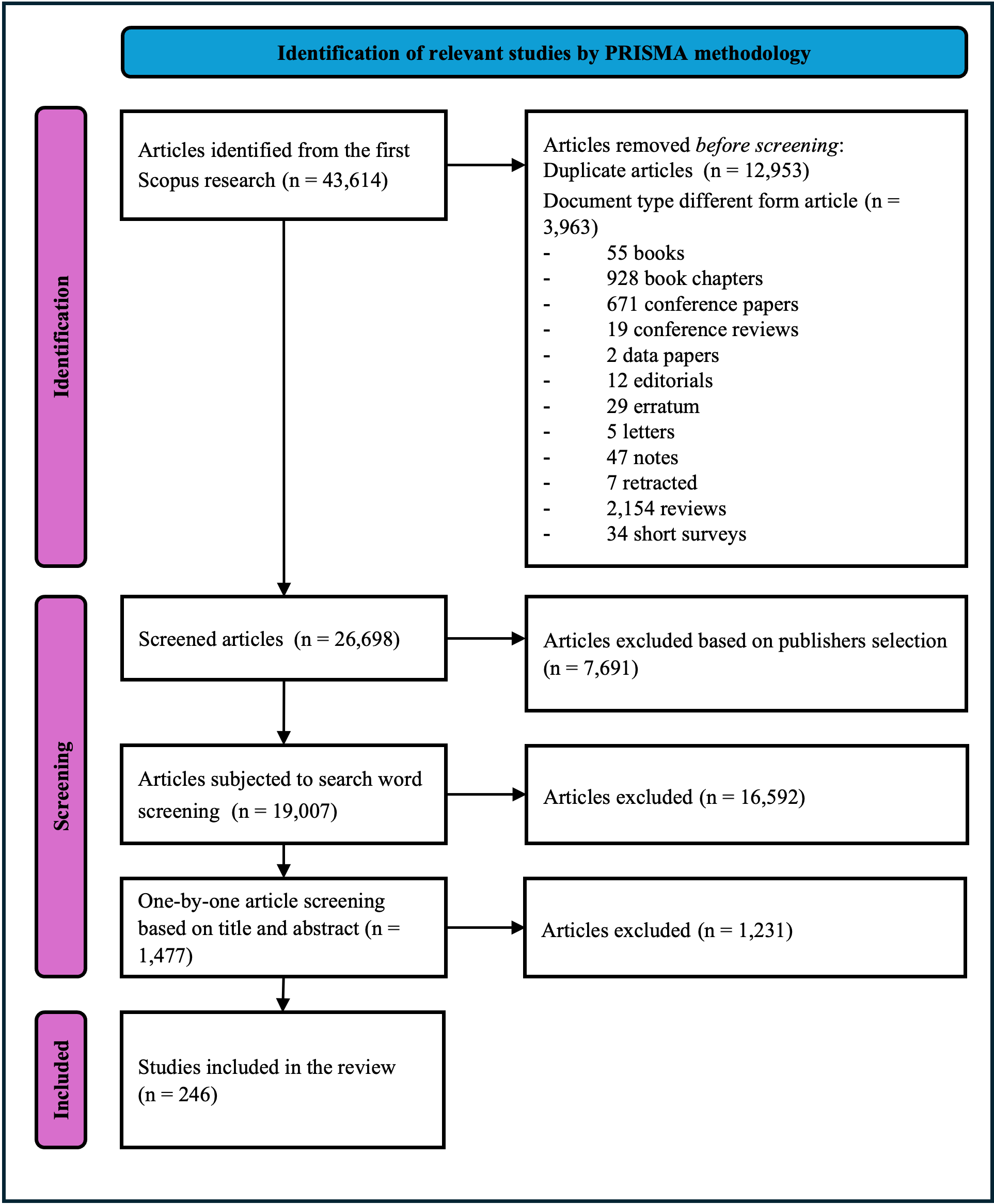


Figure S1. PRISMA flow chart.

Supplement: Multimedia component 2 [file mmc2.docx]
